# Sex-Specific Dominance of Gene Expression in Seed Beetles

**DOI:** 10.1093/molbev/msae244

**Published:** 2024-12-18

**Authors:** Philipp Kaufmann, Johanna Liljestrand Rönn, Elina Immonen, Göran Arnqvist

**Affiliations:** Department of Ecology and Genetics, Evolutionary Biology, Uppsala University, 75234 Uppsala, Sweden; Department of Ecology and Genetics, Animal Ecology, Uppsala University, 75234 Uppsala, Sweden; Department of Ecology and Genetics, Evolutionary Biology, Uppsala University, 75234 Uppsala, Sweden; Department of Ecology and Genetics, Animal Ecology, Uppsala University, 75234 Uppsala, Sweden

**Keywords:** sex-specific dominance, dominance reversal, RNA-sequencing, gene expression, sexual antagonism, *Callosobruchus maculatus*, Bruchinae

## Abstract

When different alleles are favored in different environments, dominance reversal where alternate alleles are dominant in the environment in which they are favored can generate net balancing selection. The sexes represent two distinct genetic environments and sexually antagonistic (SA) selection can maintain genetic variation, especially when the alleles involved show sex-specific dominance. Sexual dimorphism in gene expression is pervasive and has been suggested to result from SA selection. Yet, whether gene-regulatory variation shows sex-specific dominance is poorly understood. We tested for sex-specific dominance in gene expression using three crosses between homozygous lines derived from a population of a seed beetle, where a previous study documented a signal of dominance reversal for fitness between the sexes. Overall, we found that the dominance effects of variants affecting gene expression were positively correlated between the sexes (*r* = 0.33 to 0.44). Yet, 586 transcripts showed significant differences in dominance between the sexes. Sex-specific dominance was significantly more common in transcripts with more sex-biased expression, in two of three of our crosses. Among transcripts showing sex-specific dominance, lesser sexual dimorphism in gene expression among heterozygotes was somewhat more common than greater. Gene ontology enrichment analyses showed that functional categories associated with known SA phenotypes in *Callosobruchus maculatus* were overrepresented among transcripts with sex-specific dominance, including genes involved in metabolic processes and the target-of-rapamycin pathway. Our results support the suggestion that sex-specific dominance of regulatory variants contributes to the maintenance of genetic variation in fitness mediated by SA selection in this species.

## Introduction

Positive and negative selection should act to erode genetic variation, yet traits related to fitness often have genetic variances exceeding those expected under mutation–selection balance (Charlesworth and Hughes 2000; Barton and Keightley 2002; Charlesworth 2015). Antagonistic or fluctuating selection, where alternative alleles in a given locus are favored across different contexts (sexes, ontogenetic stages, seasons, environments, etc), can promote the maintenance of genetic variation. For example, sexually antagonistic (SA) selection can contribute to the maintenance of genetic polymorphism in a population (Rice 1984; Kidwell et al. 1977; Patten and Haig 2009; Connallon and Clark 2012). Intrinsic differences in reproductive strategies are often reflected in pronounced differences in phenotypic optima between the sexes for shared traits, leading to sex-specific fitness landscapes (Andersson 1994; Parker 2006; Schärer et al. 2012). However, males and females mostly rely on an overlapping set of genes that underlie traits with sex-specific fitness optima, setting the stage for intralocus sexual conflict (IaSC; Arnqvist and Rowe 2005). Quantitative genetic data, such as observations of negative genetic correlations between male and female fitness, support an important role for SA selection (Connallon and Matthews 2019).

Although sexual dimorphism is abundant across taxa (Fairbairn 2013), our understanding of how the sexes resolve IaSC to reach different phenotypes is not fully understood (Tosto et al. 2023). This problem is aggravated by the fact that genomic tools to reliably detect signatures of IaSC are lacking (Kasimatis et al. 2017), and we usually do not know which specific loci harbor SA alleles. Several potential resolutions to SA selection have been proposed, for example asymmetrical inheritance of SA genes on sex chromosomes (Rice 1984), sex-specific gene expression (Hollis et al. 2014; Veltsos et al. 2017), sex-specific posttranscriptional processes (McIntyre et al. 2006; Rogers et al. 2021), gene duplications (Bonduriansky and Chenoweth 2009; Gallach and Betrán 2011), differences in protein synthesis or posttranslational modification (Ellegren and Parsch 2007), and sex-specific dominance reversal of SA alleles (Connallon and Chenoweth 2019). Dominance reversal is of particular and general relevance. Here, each allele is dominant in the context in which it is favored, which will promote the maintenance of genetic variation under antagonistic selection because it reinforces net heterozygote advantage across contexts (Kidwell et al. 1977; Fry 2010; Wittmann et al. 2017, 2023; Connallon and Chenoweth 2019). Moreover, theory suggests that sex-specific dominance should indeed evolve under persistent SA selection (Otto and Bourguet 1999; Spencer and Priest 2016; Reid 2022; Siljestam et al. 2024) and that full dominance reversal is not required to promote the maintenance of variation, but that differences in dominance between males and females are sufficient (Siljestam et al. 2024). A growing number of empirical studies have now been able to demonstrate sex-specific dominance for a range of phenotypic traits (Barson et al. 2015; Grieshop and Arnqvist 2018; Pearse et al. 2019; Mérot et al. 2020; Geeta Arun et al. 2021; Fairbairn et al. 2023). We therefore expect a positive association between sexual antagonism and sex-specific dominance across the genome (Spencer and Priest 2016), a prediction that remains challenging to test as it requires reliably quantifying sexual antagonism and sex-specific dominance across specific genes.

While previous empirical demonstrations of sex-specific dominance involve major effect variants (Barson et al. 2015; Pearse et al. 2019; Mérot et al. 2020), a recent quantitative genetic analysis in the seed beetle *Callosobruchus maculatus* demonstrated a genome-wide pattern of dominance reversal for fitness (Grieshop and Arnqvist 2018), highlighting the potential for sex-specific dominance to partially resolve SA selection as well as its role in maintaining genetic variance associated with fitness (Connallon and Chenoweth 2019). Key phenotypic traits found to be subject to SA selection in this beetle include body size, metabolic rate, activity, and longevity (Berg and Maklakov 2012; Berger et al. 2014b; Berger et al. 2016). These traits also show dominance variation specific to (Kaufmann et al. 2021) or elevated in females (Fox et al. 2004) and the maintenance of genetic variation by SA selection (Kaufmann et al. 2023a). Sex-specific dominance variance has also been detected in fruit flies (Mukai et al. 1972) and water striders (Wolak 2013).

**Fig. 1.**
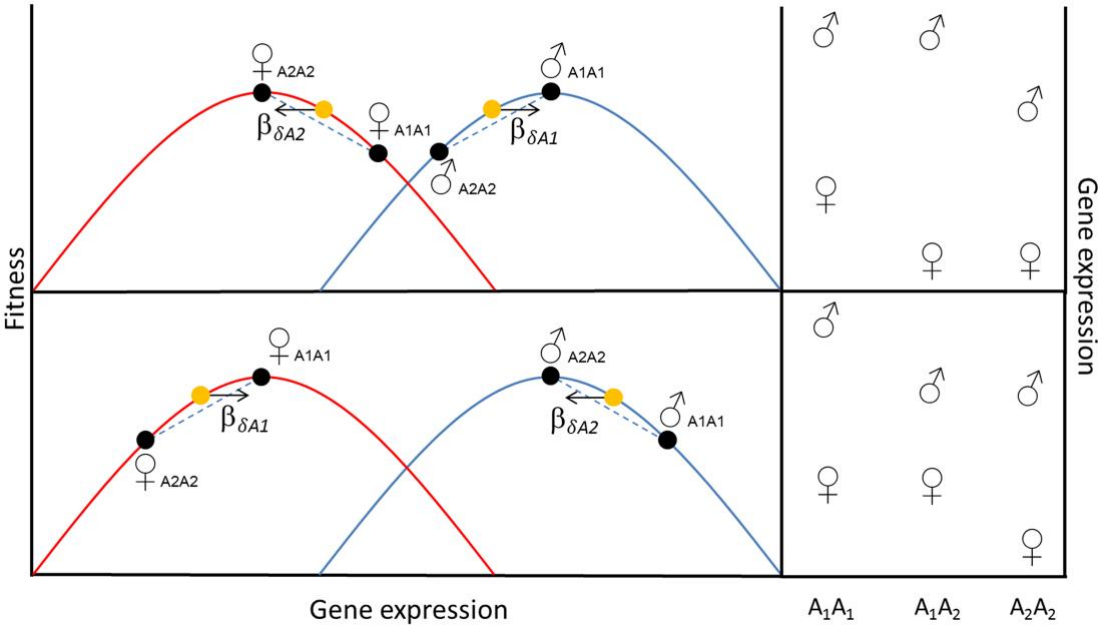
When there is segregating genetic variation in a regulatory locus A and optimal gene expression is context-specific, selection will favor context-specific dominance of gene expression (horizontal arrows). The strength of this selection will depend on the shape and location of the fitness functions. In this example, males (blue/right curves) have a higher optimal expression than females (red/left curves), and the allele A1 results in higher expression than the allele A2 in both sexes, with heterozygotes (yellow/arrowed dots) being intermediate. In the top panel, A1 is favored in males and A2 in females, and selection (β) favors dominance for A1 in males and A2 in females. In the bottom panel, A2 is instead favored in males and A1 in females, and selection favors dominance for A2 in males and A1 in females. This predicts the evolution of dominance of the allele with the highest expression in males the top scenario, and the evolution of dominance of the allele with the lowest expression in males the bottom scenario, and vice versa in females. The panels to the right show the predicted pattern of adaptive sex-specific dominance of gene expression. Note that the upper and lower cases are equivalent, but mirror-imaged, in terms of sex-specific fitness across genotypes and either could become established by e.g. novel mutations in regulatory regions or through environmentally induced shifts in sex-specific fitness functions (i.e. the adaptive landscape).

It is commonly assumed that sex-biased (SB) gene expression reflects a history of SA or sex-specific selection (see Rowe et al. [2018] for a review). This predicts that sex-specific dominance should evolve among regulatory loci affecting the expression of SB genes (Siljestam et al. 2024; Spencer and Priest 2016). Depending on precisely how regulatory variants affect sex-specific fitness, selection will promote the evolution of either an increase or a decrease of sex differences in gene expression in heterozygotes (Fig. 1). In fruit flies, studies of interspecific (Meiklejohn et al. 2014), interpopulation (Gibson et al. 2004; Mishra et al. 2024), and within-population (Puixeu et al. 2023) crosses have all documented widespread differences between the sexes in dominance of expression across genes. Yet, our general understanding of sex-specific dominance among regulatory variants segregating within populations is very limited. Here, our overall goal is to assess to what extent the architecture of gene regulation shows sex-specific dominance and to test if this is associated with SB expression. We use a population of *C. maculatus* where sex-specific dominance variance for fitness has been hypothesized to be upheld in part by sex specificity in the dominance effects of segregating regulatory variants affecting gene expression (Grieshop and Arnqvist 2018).

We make three predictions. First, unless the evolution of sex-specific dominance of gene expression is severely constrained, we expect it to be apparent given the fact that pronounced SA genetic variation has been documented in this model system (Berger et al. 2014a, 2014b, 2016; Grieshop et al. 2017). Second, if sex-specific dominance of expression evolves in response to SA selection (Fig. 1) and SB gene expression reflects SA selection, we would expect sex-specific dominance of expression to be more pronounced in genes with more SB expression. This prediction relies on SB gene expression being a valid proxy for ongoing SA selection (Connallon and Knowles 2005; Dutoit et al. 2018; Sayadi et al. 2019; Wong and Holman 2023). This prediction is muddled if, for example, sex-specific gene expression has resolved SA selection (Ellegren and Parsch 2007; Parsch and Ellegren 2013), if such loci instead experience relaxed selection (Dapper and Wade 2020), if sex bias has not had time to evolve in response to SA selection, or if dominance modifications are costly or constrained. Third, we expect those genes that show sex-specific dominance in expression to be enriched with genes affecting phenotypes known to be subject to SA selection. Here, we assess these predictions using three independent crosses among six homozygous lines derived from a population known to show SA fitness variation (Berger et al. 2014b) and sex-specific dominance reversal in fitness (Grieshop and Arnqvist 2018).

## Materials and Methods

### Study Organism and Genotype Crosses

*Callosobruchus maculatus* seed beetles are aphagous pest of legumes, and the lines studied here were kept in the laboratory under 50% relative humidity, at 29 °C, and a 12:12 light:dark cycle with black-eyed beans (*Vigna unguiculata*) as the larval host. SA fitness variation is pronounced in the population studied here (Arnqvist and Tuda 2010; Berger et al. 2014b; Berger et al. 2016; Grieshop and Arnqvist 2018) and is known to be mediated by several sexually dimorphic morphological and life history phenotypes, including body size, metabolic rate, locomotor activity, and lifespan (Berg and Maklakov 2012; Berger et al. 2014a; Berger et al. 2016; Kaufmann et al. 2021, 2023a, 2023b; Arnqvist et al. 2022). Sex differences in gene expression are large, with some 50% of all genes being markedly differentially expressed in the abdomen (Immonen et al. 2017), and genes with intermediate female bias in expression show hallmarks of balancing selection, a predicted result of long-term SA selection (Sayadi et al. 2019).

Beetles used in this study originate from a wild population collected from an agricultural field (Lomé, Togo) in 2010, which was kept in the laboratory as 41 isofemale lines (see Berger et al. 2014b). Isogenic lines were established from these isofemale lines, through single-pair full-sib inbreeding for 10 consecutive generations (see Grieshop et al. 2017). A full diallel cross experiment was used by Grieshop and Arnqvist (2018) to estimate genetic variation for fitness in each sex, which unveiled sex-specific dominance reversal for fitness. Here, we use three pairwise crosses of six of these isogenic lines to test whether the sex-specific dominance reversal seen for fitness can at least in part be due to sex-specific dominance reversal in gene expression (i.e. in transcript abundance). The six homozygous lines used showed normal productivity (i.e. no signs of inbreeding depression) and were selected based on their dominance variance rankings in both sexes (Grieshop and Arnqvist 2018) in an effort to increase the statistical power of our experiment. We created three independent pairwise crosses (I, II, and III) and studied gene expression within the isogenic lines used and in the cross between them. The parental lines are expected to be largely homozygous, while the crossed individuals will be heterozygotes for regions with fixed differences between the two isogenic lines. The heterozygote crosses were created sex reciprocally by using each line as both maternal and paternal line (i.e. dam and sire), resulting in four genotypes per cross. For convenience, we refer to the line types as genotypes (e.g. cross I: AA and BB refer to the homozygote parental line genotypes, AB and BA for the sex reciprocal heterozygote crosses; cross II: the equivalent genotypes are termed CC, DD, CD, and DC; and cross III: they are EE, FF, EF, and FE).

### RNA-Sequencing and Analyses

For each pairwise cross, we sequenced 3 biological replicates for each of the 4 genotypes and sexes, resulting in 24 samples per cross (3 × 4 × 2; i.e. 72 samples in total). For each sample, six virgin adult beetles were randomly selected and snap frozen within 24 h of their emergence from the seeds, which represents the age of peak sexual reproductive activity in both sexes. Abdomens, containing the reproductive tissue where the hallmarks of SA selection should be most manifest, of frozen beetles were collected on ice, and the six abdomens were pooled together in RNA later for 6 h at +4 °C and then stored at −80 °C until total RNA was extracted using a Qiagen RNeasy mini kit, according to the manufacturer’s protocol. cDNA libraries for the RNA-seq expression were created using the TruSeq stranded mRNA library preparation kit according to the manufacturer’s protocol, including polyA selection. NovaSeq 6000 was used to generate 150 bp paired-end sequences.

Adapter trimming was performed using fastp (v. 0.23.1; Chen et al. 2018) followed by quality control using FastQC (v 0.11.9; Andrews 2010) and MultiQC (v. 1.11; Ewels et al. 2016). RNA reads were mapped to a *C. maculatus* in-house high -uality PacBio HiFi reference genome (Kaufmann et al. 2023b) using STAR (v. 2.7.2b; Dobin et al. 2013) with splice-junction aware mapping. Duplicates were marked using Picard (Broad Institute 2019), subsequent alignment files were indexed via samtools (v. 1.12; Li et al. 2009), and RNA read counts were generated using the Subread (v. 2.0.0; Liao et al. 2014) feature count, summarizing exons per transcript.

All statistical analyses were done with R (R Core Team 2020), and plots were generated with ggplot2 (Wickham 2016). Nonrecombining, sex chromosome–linked genes were identified (Sayadi et al. 2019; Kaufmann et al. 2023b), and all analyses here were performed for autosomal and X-linked genes separately. We removed Y-linked genes prior to the analysis, after transcript mapping. We also removed transcripts that on average had <3 raw read counts per sample within each sex. The gene (transcript) expression data were inspected with DESeq2 (Love et al. 2014) using sex, genotype, and pairwise cross as fixed effects. Upon visual inspection of variance-stabilizing transformed data, we identified a few samples contaminated by mis-sexed individuals, which were removed (in total 2, 1, and 3 samples were excluded from crosses I, II, and III, respectively). Because of extensive differences in gene expression between the sexes (Immonen et al. 2017; supplementary fig. S1, Supplementary Material online), such samples can be readily identified as multivariate within-sex outliers. Variance-stabilized data were also used to create sample clustering using principal component analysis (PCA).

**Fig. 2.**
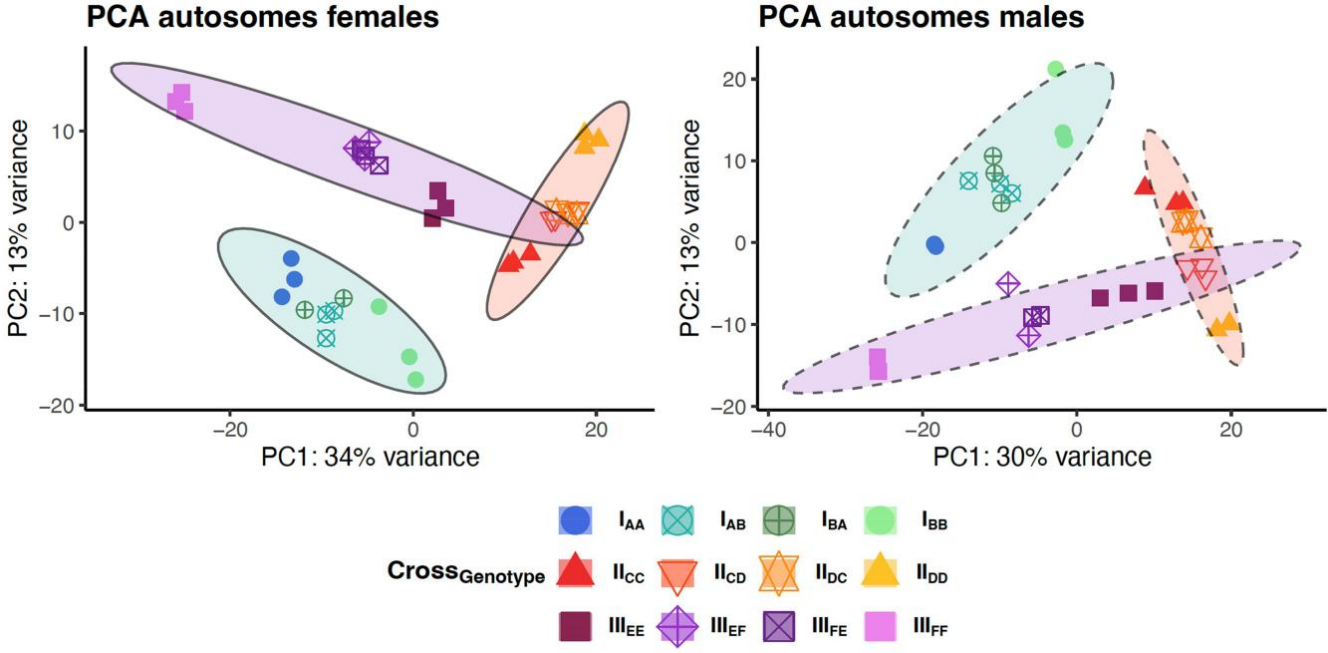
Overview of PCAs of autosomal gene expression by sex. The crosses (I, II, and III) are indicated by colored ellipses and the genotypes with symbols. Within each sex, samples cluster by cross and genotype. For both males and females, the two sex reciprocal heterozygotes (unfilled shapes) cluster together and are generally intermediate to the homozygote lines (filled shapes) within each cross (see supplementary figs. S5, S7, and S9, Supplementary Material online for each cross separately). Ellipses represent data ellipses (95% of expected data under a bivariate normal distribution).

**Fig. 3.**
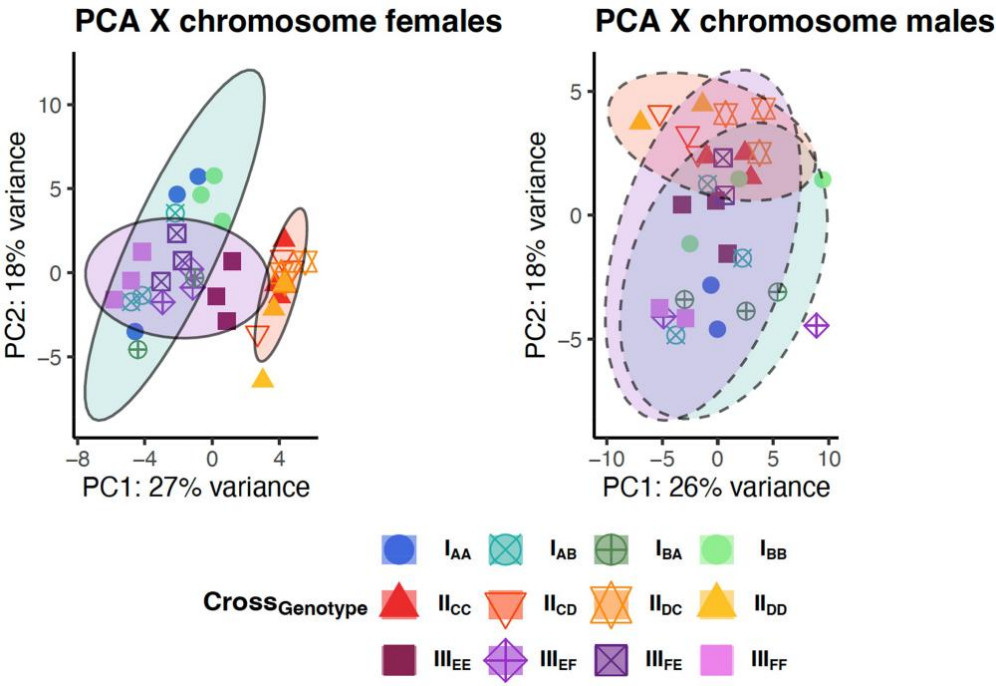
Overview of PCAs of expression of X-linked genes by sex. Male heterozygotes tend to cluster with maternal genotype (dam indicated by first letter), in line with male hemizygosity. In females, heterozygotes tend to be positioned as intermediates between the homozygote isogenic lines (see supplementary figs. S6, S8, and S10, Supplementary Material online for each cross separately). The PCAs are based on *n* = 474 X-linked transcript (out of a total of 1,841 X-linked transcripts) that passed the prefiltering steps. Ellipses represent data ellipses (95% of expected data under a bivariate normal distribution).

All analytical steps were conducted separately for each of the three crosses (except for the distance comparison across all three crosses; Figs. 2 and 3; supplementary figs. S1 and S4, Supplementary Material online) and were split by autosomal and X-linked transcripts. To identify our focal gene set, we employed a two-step filtering (see details below). First, to regard a transcript to potentially show any dominance in expression in the crosses, we required the parental homozygous lines to show a significant difference in transcript abundance. The logic here is that only transcripts with a difference in expression between the two parental homozygotes can show dominance (thus excluding any cases of pure overdominance or underdominance where the two homozygotes are identical). Second, for transcript expression to reflect consistent SA selection, sex bias in expression should be similar in the two homozygous parental lines.

To estimate differential gene expression (as a log_2_-fold change [LfC]) between males and females in homozygotes (e.g. AA and BB), we used the DESeq2 package (Love et al. 2014). Additionally, we used DESeq2 to calculate variance-stabilized transformed expression matrices that were used for the PCA. We then proceeded to include all heterozygote crosses and used total read count analysis from the R package rxSeq (Zou et al. 2014) to jointly estimate sex-specific additive as well as dominance allelic effects and their interaction with sex, while accounting for potential parent-of-origin effects. The dominance coefficient (δ) estimated from the rxSeq model represents the log of the observed heterozygote gene expression (e.g. $AB_{O}$) divided by the expected heterozygote gene expression under complete additivity (e.g. $AB_{E}=\;\left(\frac{AA+BB}{2}\right)$, the mean between the parental homozygotes), i.e. $e^{\delta }=\frac{AB_{O}}{AB_{E}}$. Hence, if gene expression is completely additive ($AB_{O}=AB_{E}$), then δ=0, and if gene expression is dominant ($AB_{O}>AB_{E}$) or recessive ($AB_{O}<AB_{E}$), then *δ* will be positive or negative, respectively. To clarify, for each gene, the homozygous line with higher expression was here designated as BB such that *δ* always refers to the observed dominance of the regulatory allele that encodes for a relatively high expression when in a homozygous state (in both sexes). To call sex-specific dominance in a transcript within our focal set (see below), we required a significant sex × dominance interaction in these models. We note that rxSeq essentially averages over the two sex reciprocal heterozygote crosses when estimating the dominance coefficients. In our case, this is motivated by the fact that very few transcripts showed sex-consistent parent-of-origin effects in transcript abundance (fraction of focal transcripts showing a significant parent-of-origin effect in both male and female samples in the three crosses were as follows: I: 0%; II: 1.1%; and III: 2.2%). To account for multiple testing, we calculated the *q*-values using the R package qvalue (Storey et al. 2024) with a 0.05 *q*-value cutoff throughout.

To identify the focal set of transcripts, we applied the two-step filtering referred to above as follows. First, within each cross, using only data on the parental homozygous lines, we removed transcripts that did not show a significant differential expression between the parental lines (e.g. AA vs. BB). Most such loci are likely not biallelic within the cross and thus not informative of dominance. This removed 6,234, 6,019, and 4,783 autosomal transcripts from crosses I, II, and III, respectively. Second, we retained only genes showing sexually concordant differences in expression between the homozygous parental genotypes, by removing transcripts that had a significant sex-by-parental line interaction in models including only parental lines. Such loci with sex-specific genetic effects in homozygotes are likely not constrained by sex-correlated additive effects and therefore have no or a much-reduced potential for SA selection. This removed another 2,347, 2,087, and 3,534 transcripts from crosses I, II and III, respectively, leaving 3,407, 3,412, and 3,534 to form our focal set of transcripts for further analyses. The dominance correlation between the sexes was subsequently estimated using a type II regression in the R package lmodel2 (Legendre 2018).

Gene ontology enrichment analyses for biological processes were made using topGO (Alexa and Rahnenfuhrer 2024), based on genes passing the filtering steps in each cross as the reference set, to test whether any functional categories of genes are associated with sex-specific dominance. Significant terms identified by the GO enrichment analyses were clustered based on their hierarchical associations and visualized with the R package ViSEAGO (Brionne et al. 2019).

## Results

### Overall Variation in Expression Across Genotypes and Sexes

PCAs revealed that the largest signal of differential expression in the autosomal transcript expression was due to sex differences, as expected, with the samples clustering by sex on PC1 that explained over 85% of the variance in our data (supplementary fig. S1, Supplementary Material online). Splitting the samples by sex revealed clear clustering of samples by cross and genotype (Fig. 2), consistent with substantial genetic variation in gene regulation. Within each cross, sample distance (PC1 and PC2) based on autosomal expression between the parental homozygous isogenic lines was similar for female and male samples, implying that on average the genotype differences in gene expression are similar in magnitude in the sexes. In both sexes, cross III showed the largest differences in autosomal transcript expression among the genotypes, cross I was intermediate, and cross II showed the lowest level of genotype differences (Fig. 2). As expected, within each cross, overall autosomal transcript expression in heterozygotes was intermediate to the parental homozygous isogenic lines. Inspection of the pair-wise correlations in transcript abundance (Spearman's rho − 1) between samples across all autosomal transcripts largely confirmed these interpretations and showed that the largest difference in gene expression between parental homozygous lines occurred in cross III and the lowest in cross II (supplementary figs. S2 and S3, Supplementary Material online). Differences across samples within each cross are detailed in supplementary figs. S2, S3, S5 to S10, Supplementary Material online.

X-linked transcript expression similarly showed clustering of samples by sex along PC1 (supplementary fig. S4, Supplementary Material online), but there were less differences between the crosses or genotypes within each cross (Fig. 3). Note that the amount of data is restricted due to much fewer X-linked than autosomal transcripts. Nevertheless, within each cross, heterozygous females clustered in between the parental homozygotes (supplementary figs. S6, S8, and S10, Supplementary Material online). In male heterozygote crosses, X-linked transcripts cluster based on the mother's genotype in males in crosses II and III, while the pattern was less clear in cross I (supplementary figs. S6, S8, and S10, Supplementary Material online). Given that males are hemizygous for the X, no clustering by paternal genotype is expected for males for X-linked transcripts. In summary, clustering of genotypes based on their differences in gene expression followed expectations well and revealed a larger difference between homozygous lines in crosses III and I.

### Identification of Dominance in Gene Expression

Our analytical workflow to test for sex difference in dominance for autosomal transcripts included a priori criteria for a transcript to be considered further in the analysis of sex-specific dominance, including a significant and sexually concordant differential expression between the parental homozygous genotypes within each cross. Similar numbers of autosomal transcripts fulfilled these criteria in the three crosses (3,407, 3,412, and 3,534 for I, II, and III, respectively), by showing both a significant differential expression between the parental homozygous lines and no significant sex-by-parental line interaction among the parental homozygous lines. Across these focal sets, approximately half of the transcripts were private to each cross (48%, 51%, and 48%), and only ∼11% of the transcripts were shared among all three crosses (supplementary fig. S11, Supplementary Material online). This strongly suggests that the parental isogenic lines captured distinct regulatory variants from the original source population, an interpretation strengthened by the clear clustering of samples by cross (Fig. 2).

**Fig. 4.**
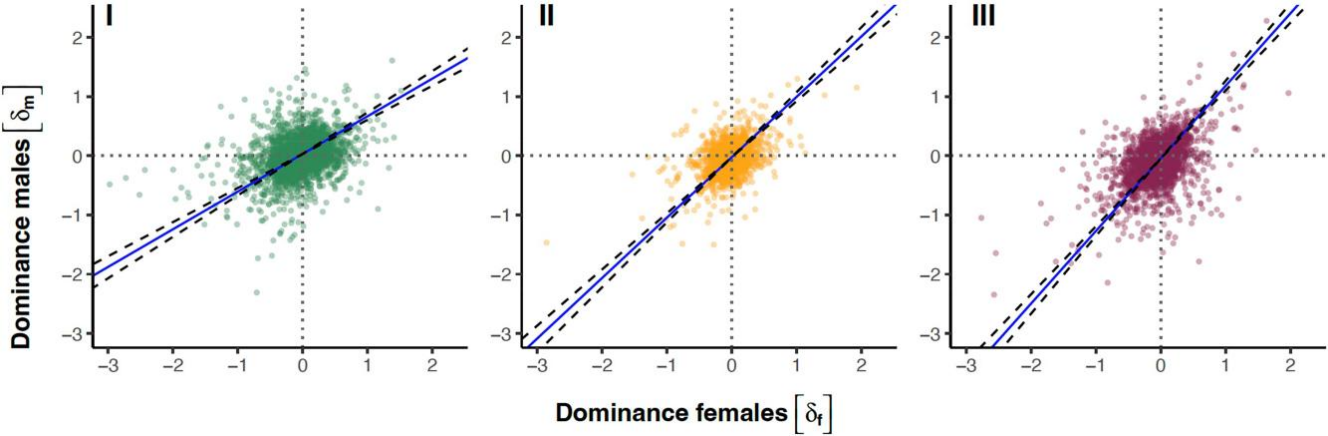
The female and male dominances (*δ*) of transcript expression were positively correlated in all three crosses (*n* = 3,407, 3,412, and 3,534 transcripts for crosses I, II, and III, respectively). Shown are slopes of major axis regressions (solid line) with 95% credible intervals (dashed lines). Positive *δ* indicates that higher transcript expression is dominant, *δ* of zero indicates that transcript expression is additive, and negative *δ* indicates that higher transcript expression is recessive in a given sex.

**Fig. 5.**
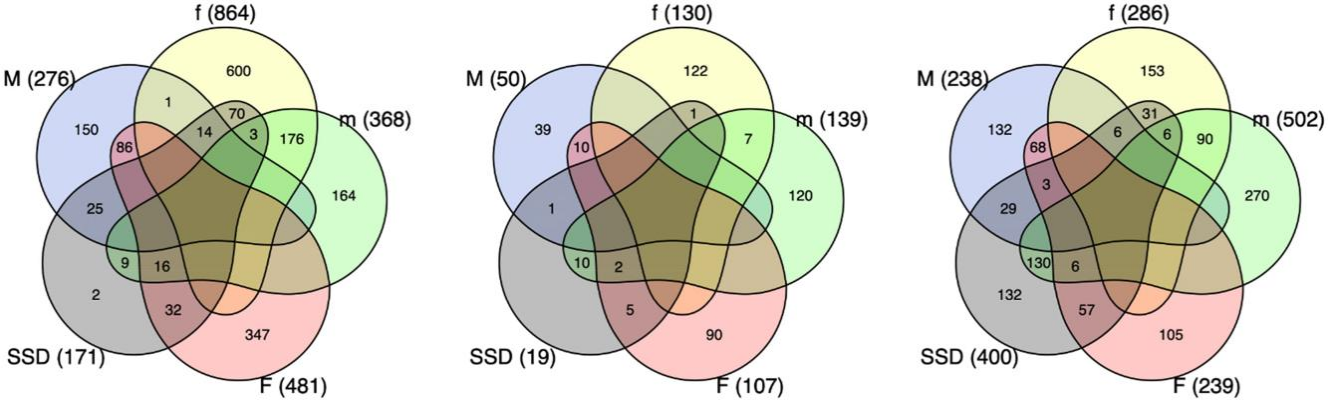
Venn diagrams of transcripts with nonadditive expression. Shown are the numbers of transcripts with significant dominance in males (M) and females (F), significant recessivity in males (m) and females (f), and significant sex-specific dominance (SSD), given separately for crosses I (left), II (center), and III (right). Few transcripts with SSD showed significant dominance in one sex and significant recessivity in the other (I: 30, II: 2, and III: 12). Instead, most transcripts with SSD showed dominance or recessivity in one sex and no significant deviation from additivity in the other.

**Fig. 6.**
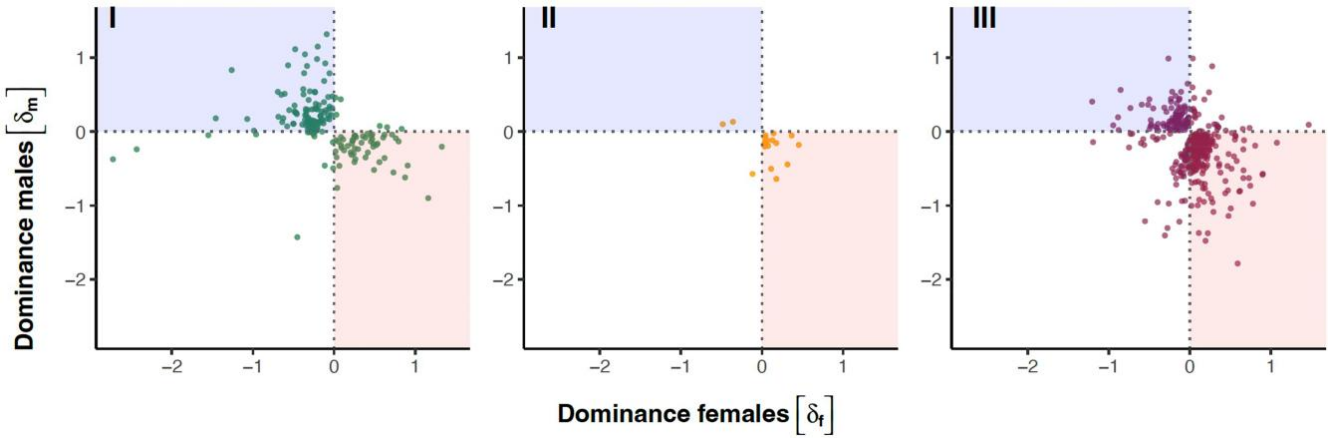
Distribution of the dominance coefficient (*δ*) for those transcripts that show a significant sex × dominance interaction (*q* = 0.05). Across all three crosses, the majority of these transcripts show dominance reversal, where a transcript is dominant in females but recessive in males (lower/right quadrant highlighted in red) or dominant in males and recessive in females (upper/left quadrant highlighted in blue). Positive *δ* indicates that higher transcript expression is dominant and a negative *δ* indicates that higher transcript expression is recessive.

Across all transcripts in our focal sets, there was a medium strong correlation between the dominance parameter in males and females in all three crosses (type II regression: cross I: slope = 0.65, 95% confidence interval [CI] [0.59 to 0.72], *r* = 0.33, *P* < 0.0001; cross II: slope = 1.02, 95% CI [0.95 to 1.10], *r* = 0.42, *P* < 0.0001; and cross III: slope = 1.22, 95% CI [1.14 to 1.31], *r* = 0.44, *P* < 0.0001; Fig. 4) suggesting that dominance effects of variants affecting expression are often, but not always, concordant in males and females. In line with this, our analyses uncovered in total 586 transcripts with significant sex differences in dominance (i.e. a significant sex × dominance interaction), although the numbers differed markedly among the three crosses (171, 19, and 400 for I, II, and III, respectively). There was very little overlap in terms of transcript identities (supplementary fig. S11, Supplementary Material online), again suggesting that distinct allelic variants were exposed in the different crosses. The number of significant cases in different crosses is well aligned with the previous analyses, which showed the greatest distance between parental homozygous genotypes in cross III and the smallest in cross II (Fig. 2, supplementary fig. S2, Supplementary Material online). In general, many transcripts that showed sex difference in dominance were significantly nonadditive in one sex but not in the other (Fig. 5). Fewer transcripts showed a complete significant reversal of dominance, where a transcript showed significant dominance in one sex and significant recessivity in the other sex. We note, however, that while sex specificity was often not reflected in a transcript being significantly dominant in one and significantly recessive in the other sex, the vast majority of transcripts with a significant sex × dominance interaction did show a reversal in direction (i.e. occupied lower-right or upper-left quadrant in Fig. 6).

### Sex-Specific Dominance and Sex Bias

The number of focal genes showing greater versus lesser SB expression in heterozygotes relative to homozygotes (i.e. top-right panel vs. lower-right panel in Fig. 1) was fairly similar overall, but this proportion differed in the three crosses (supplementary fig. S12, Supplementary Material online). Among the subset of focal transcripts that showed significant sex-specific dominance, the proportion that showed greater SB expression in heterozygotes was lower than 0.5 in cross I (0.15; χ^2^_1_ = 85.6, *P* < 0.001), indistinguishable from 0.5 in cross II (0.42; χ^2^_1_ = 0.47, *P* = 0.492), and higher than 0.5 in cross III (0.57; χ^2^_1_ = 7.29, *P* = 0.007). Overall, transcripts showing sex-specific dominance thus involved cases of both greater (*n* = 260) and lesser (*n* = 330) SB in expression among heterozygotes, though the latter was more common (χ^2^_1_ = 8.30, *P* = 0.004).

**Fig. 7.**
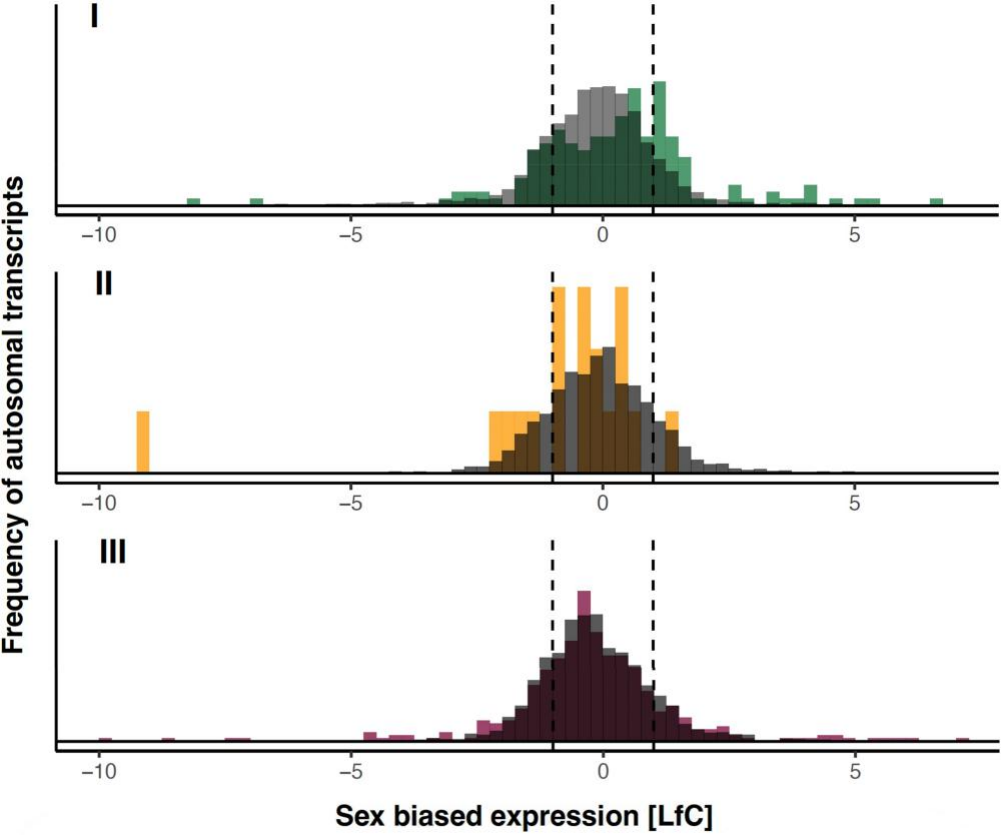
Distribution of sex bias in expression of autosomal genes. Density histograms of all focal transcripts (gray), that serve as reference distributions, and those with significant sex-specific dominance (*q* = 0.05; colored) in the three independent crosses (I to III). The *x* axis shows SB expression as a LfC. Negative values mean that a transcript is male biased in expression and positive values mean that it is female biased. Dashed lines indicate a 2-fold difference between the sexes in transcript abundance. In crosses I and III, which showed most sex-specific dominance, transcripts with strong sex bias in expression were enriched with genes showing sex-specific dominance in expression.

Transcripts showing sex-specific dominance showed significantly higher SB expression compared with transcripts in our focal gene set that did not show sex-specific dominance, in two out of our three crosses (Fig. 7; directional Kolmogorov–Smirnov tests of equality of the distributions of absolute values of SB in the two sets of transcripts; cross I: *D*^+^ = 0.18, *P* < 0.0001; cross II: *D*^+^ = 0.09, *P* = 0.755; and cross III: *D*^+^ = 0.07, *P* = 0.040). A visual inspection of the results (Fig. 7) suggests that this was to a large extent due to strongly SB genes being overrepresented among genes with sex-specific dominance. Further, in cross I, the proportion of female-biased genes showing sex-specific dominance was also significantly higher than among male-biased genes (Fisher's exact test; cross I: *P* < 0.0001). In crosses II and III, however, these proportions did not differ significantly (Fisher's exact tests; cross II: *P* = 0.232, cross III: *P* = 0.682; Fig. 7).

### Gene Ontology Enrichment of Sex-Specific Dominance Genes and X-Linkage

**Fig. 8.**
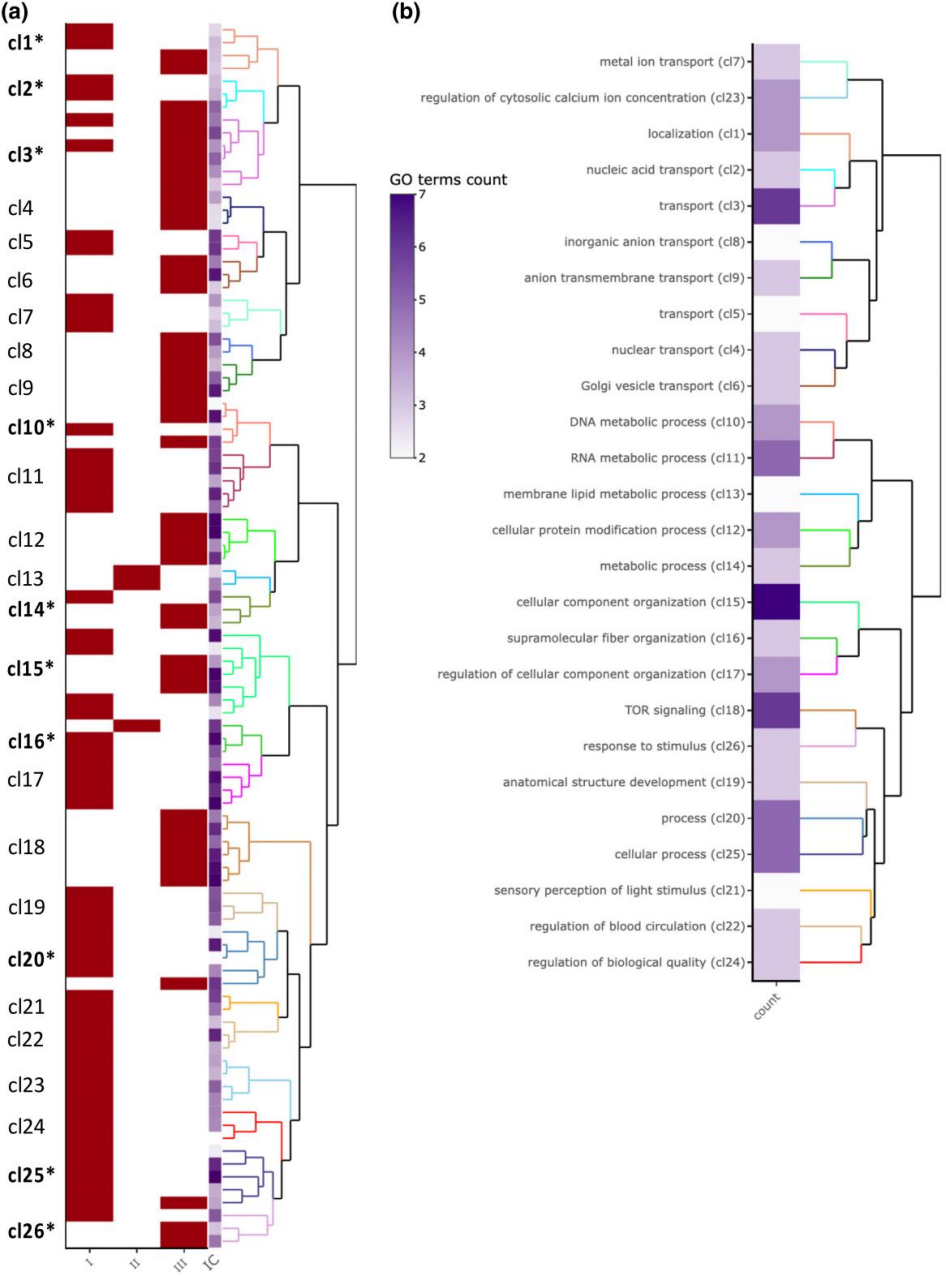
Gene ontology enrichment for transcripts with a significant sex × dominance interaction compared with all genes that passed the filtering, calculated separately for each cross. a) GO term dendrogram based on Wang's semantic similarity distance and ward.D2 clustering. Terms highlighted in red are significantly enriched in the respective cross (I, II, or III). Each GO term cluster is indicated in a different color in the dendrogram. Clusters highlighted in bold and with an asterisk have significant enrichment in more than one cross. Note that the reference set of genes (i.e. those that passed the filtering) used to calculate enrichment is different for the three crosses (supplementary fig. S11, Supplementary Material online). b) Dendrogram based on best-match average semantic similarity and ward.D2 aggregation, showing the first common GO term ancestor for each cluster from a) and the number of enriched GO terms within each cluster in purple.

We identified a series of enriched GO terms among transcripts with significant sex × dominance interaction. Interestingly, despite the restricted overlap in the identity of these transcripts between the crosses (supplementary fig. S11, Supplementary Material online), a number of higher hierarchical terms overlapped among enriched GO terms between crosses (Fig. 8). This is consistent with some functional similarity in genes with sex-specific dominance in expression. Most of these overlapping terms describe broad generic functions such as organization, location, transport, and cellular process, but they also included functions associated with known SA phenotypes such as various metabolic processes and *target of rapamycin* (TOR) signaling. Closer inspection of genes with sex-specific dominance within each of the three crosses (supplementary fig. S13 to S15, Supplementary Material online) again not only identified enrichment for a number of general functions but also showed significant enrichment for a series of more specific functions previously inferred to be associated with SA selection, such as a variety of metabolic processes (Berger et al. 2014a; Arnqvist et al. 2017, 2022), TOR signaling (Kaufmann et al. 2023b), mitochondrial function (Dowling et al. 2007; Immonen et al. 2016), immune function (Bagchi et al. 2021), sexual reproduction (Sayadi et al. 2019), and DNA repair (Baur and Berger 2020; Koppik et al 2023). Detailed GO term enrichment and associated genes is given in supplementary table S1, Supplementary Material online. To assess whether the functional terms identified are specifically associated with sex-specific dominance or dominance per se, we also tested for GO term enrichment among transcripts with sexually concordant dominance. This comparison (supplementary figs. S13 to S15, Supplementary Material online) showed that terms enriched in genes with sex-concordant and sex-specific dominance were largely nonoverlapping.

We found no significant difference between the proportion of genes with significant female nonadditivity between autosomal and X-linked genes in either cross (Fisher's exact test: cross I: 95% CI [0.61 to 1.49], *P* = 0.827, cross II: 95% CI [0.33 to 82.5], *P* = 0.719, and cross III: 95% CI [0.71 to 3.58], *P* = 0.337). Overall, a restricted number of X-linked transcripts showed female nonadditivity (i.e. significant dominance or recessivity) in any of the crosses: 31, 1, and 8 transcripts in crosses I, II, and III, respectively (supplementary fig. S16, Supplementary Material online). There was no clear overrepresentation of genes with female nonadditivity in SB or unbiased genes (supplementary fig. S17, Supplementary Material online). Note that the low number of nonadditive X-linked genes does not allow for a statistical test with reasonable power when the data are split by SB expression.

## Discussion

Sex-specific dominance modifiers are predicted to evolve for alleles under persistent SA selection (Spencer and Priest 2016; Siljestam et al. 2024). Here, we assessed the pattern of sex-specific dominance in gene expression in a population of an insect where SA genetic variance (Berger et al. 2014b) and sex-specific dominance reversal for fitness have been suggested to reflect sex-specific dominance in gene expression (Grieshop and Arnqvist 2018). Overall, our findings support a role for sex-specific dominance of gene expression in the maintenance of SA genetic variation in this population. In particular, we found support for our three general predictions, sex-specific dominance in gene expression occurred in many genes, was overrepresented in genes with SB expression and was associated with a series of known SA phenotypes. Below, we discuss each of these three findings.

The allelic effects of variants affecting gene expression were additive on average, but the correlation between dominance across the sexes was less than perfect (Fig. 4), showing that the architecture of gene regulation is not entirely shared between the sexes. Importantly, our analyses uncovered 586 transcripts (8% of all focal transcripts) with significant sex-specific dominance, many of which showed dominance reversal between the sexes (Fig. 6). Others tended to show additivity in one sex and significant dominance/recessivity in the other, a pattern of inheritance, which in theory can also promote the maintenance of genetic variation (Siljestam et al. 2024). Interestingly, we found that the specific transcripts showing sex-specific dominance were only rarely shared across our three crosses, and the patterns we uncovered were sometimes distinct across crosses. A large effect of genetic background on the sex-specific regulatory architecture has been documented also in *Drosophila* (Glaser-Schmitt et al. 2021; Puixeu et al. 2023). This likely reflects the complex and polygenic nature of gene-regulatory networks (Glaser-Schmitt et al. 2018) but may also in part be due to segregating polyallelic polymorphism in regulatory regions (Siljestam et al. 2024).

Similar recent efforts in *Drosophila* have also demonstrated significant sex-specific dominance in gene expression in reproductive tissues, based on comparisons between (25%; Mishra et al. 2024) and within (7% to 11%; Puixeu et al. 2023) populations. These studies, however, analyzed allelic imbalance in heterozygotes so were restricted to genes showing fixed differences in coding regions among parental homozygous lines. These findings, in combination with the fact that SB in gene expression is pervasive, suggest that sex-specific dominance of regulatory elements may play an underappreciated role in the maintenance of SA genetic variation. In fact, there are reasons to believe that the significance of dominance reversal in expression may extend to contexts other than the sexes (Wittmann et al. 2017; Johnson et al. 2023), and a few studies provide support for such a role. For example, Chen et al. (2015) demonstrated widespread allelic effects on gene expression that alternated between being dominant and recessive depending on the thermal environment in fruit flies, while Posavi et al. (2014) observed beneficial dominance reversal for loci associated with salinity tolerance in the copepod *Eurytemora affinis*.

Previous studies of *Drosophila* have found that sex specificity in dominance of gene expression is caused mainly by *cis*-regulatory variation (Meiklejohn et al. 2014; Mishra et al. 2024). Our experiments were not designed to assess the relative roles of *cis*- and *trans*-regulatory factors, which precludes conclusion in this regard other than that both may be involved in *C. maculatus*. This said, we suggest that the fact that transcripts showing sex-specific dominance were rarely shared between our replicate crosses is perhaps difficult to reconcile with a more global role for major sex-specific *trans*-regulatory factors.

The X chromosome is a potential hotspot for SA alleles, and it has been suggested that both male and female beneficial alleles should accumulate on the X depending on dominance relationship in females (Rice 1984). One could therefore expect the X to harbor more SA transcripts that show nonadditivity in females compared with the autosomes. We did not find evidence for this in any of the crosses. This aligns with previous work showing a lack of enrichment of candidate SA variants on the X (Sayadi et al. 2019), and it suggests that the X is not a hotspot for genes showing female nonadditivity in expression in *C. maculatus*. We do note, however, that the low number of X-linked transcripts with female nonadditivity restricts the statistical power of these analyses. Further, the X chromosome assembly is not complete, and it remains possible that yet undetected regions of the X show a different pattern.

We found support for the prediction that genes with more SB expression should be significantly enriched with genes showing sex-specific dominance in expression, in two of three crosses (Fig. 7). The direction of SB in gene expression is often assumed to be a viable proxy for the direction of SA selection and optimal expression for each sex (Rice 1984; Connallon and Knowles 2005; Ellegren and Parsch 2007; but see Parsch and Ellegren 2013; Immonen et al. 2017). This does, however, not necessarily translate into predictions concerning the directionality of adaptive dominance reversal based on SB expression. In fact, segregating SA genetic variation in regulatory loci could in theory favor either dominance or recessivity of the allele encoding for higher expression in the sex where higher expression is favored (Fig. 1). The fact that we found little consistent directionality in this regard, because sex-specific dominance did not generally result in greater SB in heterozygotes compared with homozygotes (supplementary fig. S12, Supplementary Material online) across our crosses, suggests that the direction of SB is not a strong predictor of the direction of selection for sex-specific dominance in regulatory regions under SA selection in *C. maculatus*.

*Callosobruchus maculatus* is a well-established model organism to study sexual conflict, and several key life history phenotypes are known to be under SA selection, including body mass, lifespan, metabolic rate, growth, locomotion, immune function, mitochondrial function, DNA repair, and gene expression (Dowling et al. 2007; Berg and Maklakov 2012; Berger et al. 2014a, 2016; Immonen et al. 2016; Immonen et al. 2017; Sayadi et al. 2019; Baur and Berger 2020; Bagchi et al. 2021; Arnqvist et al. 2022; Koppik et al. 2023). Our main aim was to assess whether the gene-regulatory architecture shows sex-specific dominance, and thus is permissive for the maintenance of SA genetic variation in this species, rather than to identify candidate SA genes. Although functional enrichment analyses should be interpreted with some caution, we note that genes with sex-specific dominance did show enrichment for a series of functions associated with known SA phenotypes in this species. A particularly interesting and more specific finding is the enrichment of the TOR growth pathway in cross III, where no less than six transcripts involved in TOR signaling showed sex-specific dominance. We have recently found that the TOR pathway is likely central in sexual antagonism over body size in *C. maculatus*, with males carrying an extra copy of the otherwise autosomal TOR on the Y chromosome (Kaufmann et al. 2023b). We have also documented SA selection on body size (Rankin and Arnqvist 2008; Arnqvist and Tuda 2010) and further discovered that dominance genetic variation in body size is, in fact, sex-specific (Kaufmann et al. 2021; Kaufmann et al. 2023a). The fact that autosomal genes in the TOR pathway show sex-specific dominance in gene expression is consistent with these findings and suggests that multiple mechanisms have evolved to mitigate sexual conflict over life history traits associated with TOR signaling in this species.

A third observation offers further, albeit indirect, support for an enrichment of SA effects among genes with sex-specific dominance in *C. maculatus*. Theory predicts that context-specific dominance should promote allelic polymorphism, by strengthening net balancing selection through marginal overdominance in loci experiencing conflicting selection (Wittmann et al. 2017, 2023; Connallon and Chenoweth 2019; Siljestam et al. 2024). Recently, Siljestam et al. (2024) employed population resequencing data and showed that allelic diversity was markedly and significantly elevated among the set of 586 genes that showed sex-specific dominance here. This finding is consistent with a role for sex-specific dominance in the maintenance of genetic variation.

## Conclusion

In line with the tenet that SA selection is associated with sex-specific modifiers of dominance, we identified 586 genes that showed sex-specific dominance effects in their expression in a species showing SA genetic variation and where sex-specific dominance reversal in fitness has previously been documented. Moreover, genes with more SB expression were overrepresented among such genes, and a series of functions involved in phenotypic traits known to be under SA selection in *C. maculatus* were enriched among genes with sex-specific dominance. Theory and previous work show that SA genetic variation in fitness can be maintained in part by net balancing selection promoted by sex-specific dominance reversal (Grieshop and Arnqvist 2018; Connallon and Chenoweth 2019; Siljestam et al. 2024). Our present findings suggest that this is at least in part due to sex-specific dominance effects in regions that affect gene expression, in line with the observation that the genes here identified as showing sex-specific dominance also show elevated within-population allelic diversity (Siljestam et al. 2024). One implication of our results is therefore the possibility that the targets of SA selection may commonly be various noncoding regions with sex-specific effects on gene expression (Mishra et al. 2024). More generally, recent data based on genome scans (Bergland et al. 2014; Machado et al. 2021; Rudman et al. 2022) have revived the possibility that spatially or temporally antagonistic selection may result in widespread balancing selection through environment-specific dominance (Wittmann et al. 2017, 2023; Johnson et al. 2023). Our work can serve as a reminder of the fact that there is no reason to expect such effects to be restricted to protein-coding regions of the genome.

## Supplementary Material

msae244_Supplementary_Data

## Data Availability

All raw RNA-Seq data (72 samples) have been deposited in FASTQ format in ENA sequence read repository under the BioProject accession number PRJEB70958.
