# Supplementary material for: Sex-Specific Dominance of Gene Expression in Seed Beetles
Source: Mol Biol Evol. 2024 Dec 18;41(12):msae244. doi: 10.1093/molbev/msae244 (PMC11653567; doi:10.1093/molbev/msae244)
Supplement: msae244_Supplementary_Data [file msae244_supplementary_data.zip › SI_SSDR_2024_v4.pdf]

Supporting Information

Sex-specific dominance of gene expression in seed beetles

Philipp Kaufmann[1*], Johanna Liljestrand Rönn[2], Elina Immonen[1] & Göran Arnqvist[2]

[1] Department of Ecology and Genetics, Evolutionary Biology, Uppsala University, Norbyvägen 18D, 75234 Uppsala, Sweden.
[2] Department of Ecology and Genetics, Animal Ecology, Uppsala University, Norbyvägen 18D, 75234 Uppsala, Sweden.

Contents:

Figures S1 – S17

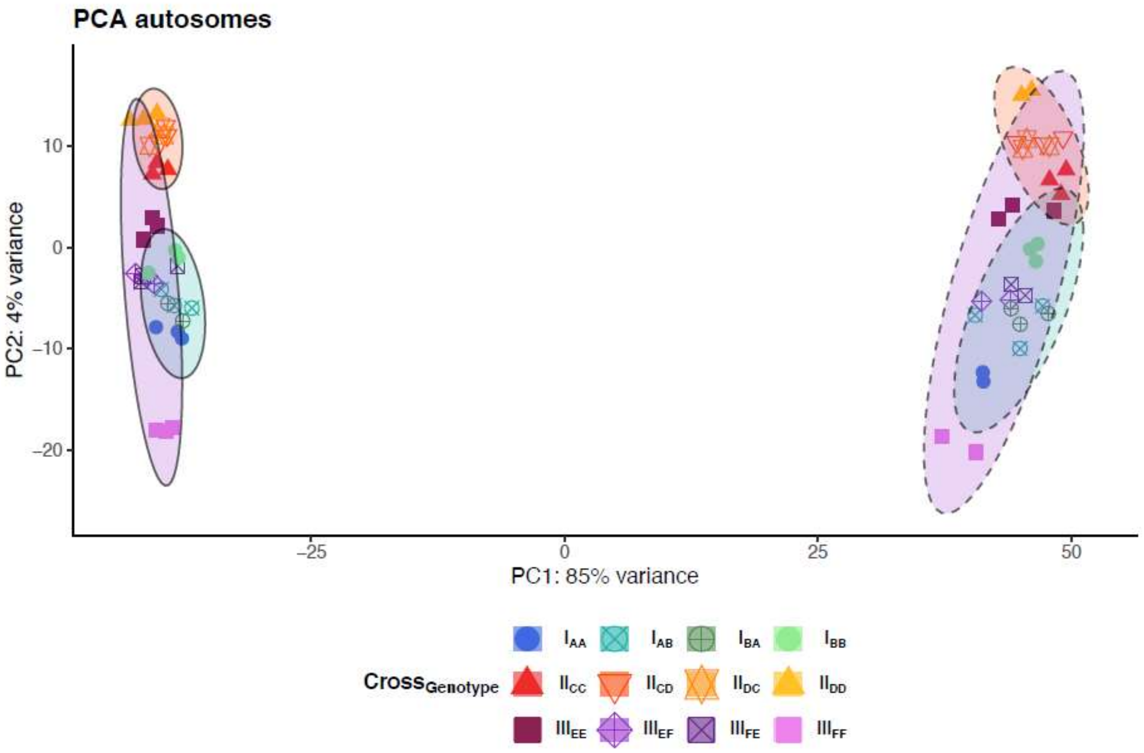

Fig. S1| Overview of autosomal gene expression (PCA). The colors and ellipsoids indicate the three crosses (I, II & III). Overall, the samples cluster well by sex along PC1 (i.e. female samples in solid line ellipsoids, male samples as dashed line ellipsoids), explaining a large part of variance in expression (85%). The PCA is based on N = 10762 autosomal transcripts (out of a total of 37673 autosomal transcripts) that passed the pre-filtering the three crosses. Ellipses represent data ellipses (95% of expected data under a bivariate normal distribution).

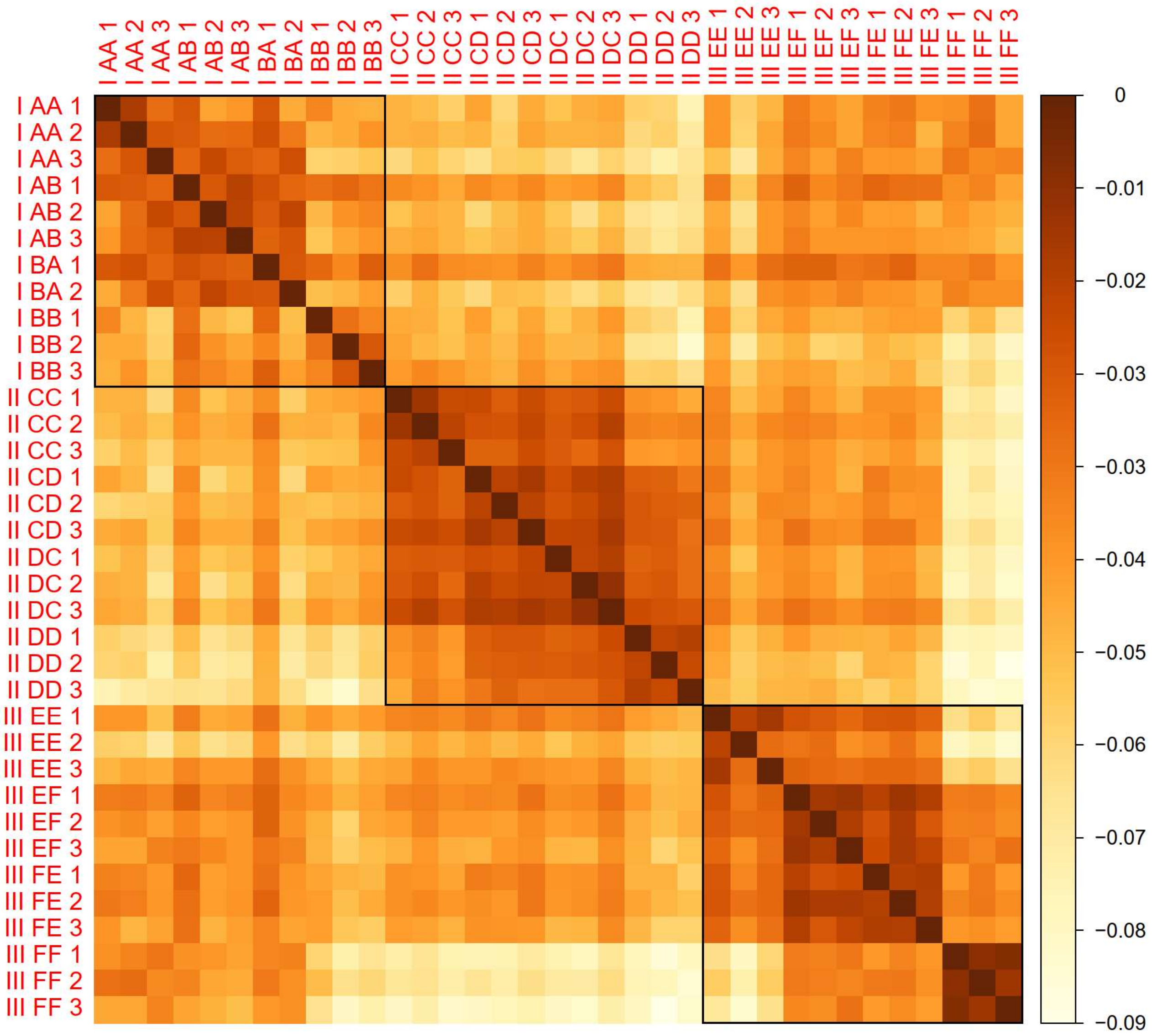

Fig. S2| Correlation matrix (Spearmans' rho - 1) of gene expression across all female samples. The figure shows the correlation in transcript abundance across all autosomal transcripts. Black squares enclose the three crosses and letters denote cross type and the specific genotype cross in each sample. Note, for example, that the largest difference in gene expression between parental homozygous lines occurred in cross III (i.e., EE vs. FF) and the smallest in cross II (i.e., CC vs. DD).

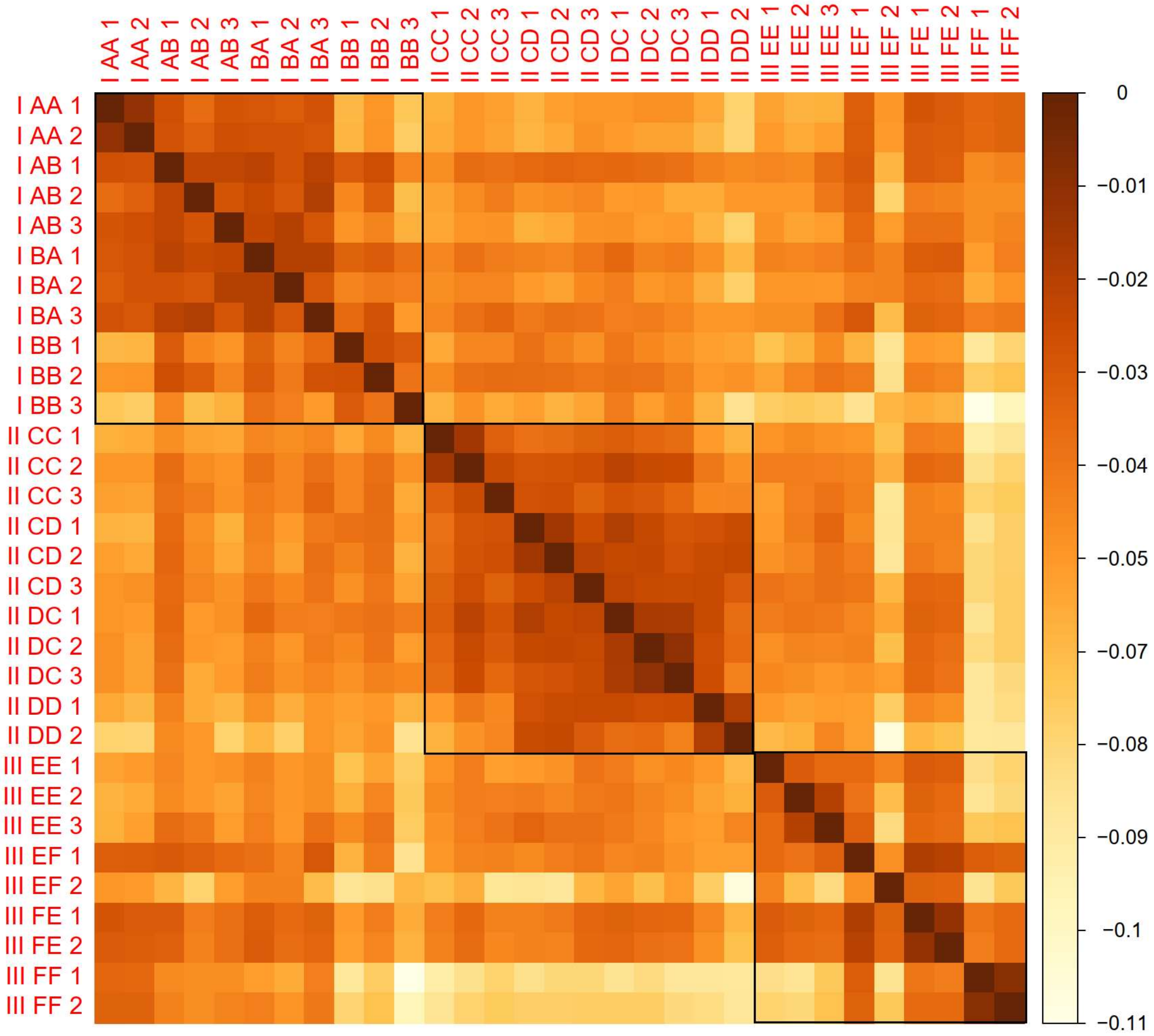

Fig. S3| Correlation matrix (Spearmans' rho - 1) of gene expression across all male samples. The figure shows the correlation in transcript abundance across all autosomal transcripts. Black squares encloses the three crosses and letters denote cross type and the specific genotype cross in each sample. As in female samples (Figure S2), the largest difference in gene expression between parental homozygous lines occurred in cross III (i.e., EE vs. FF).

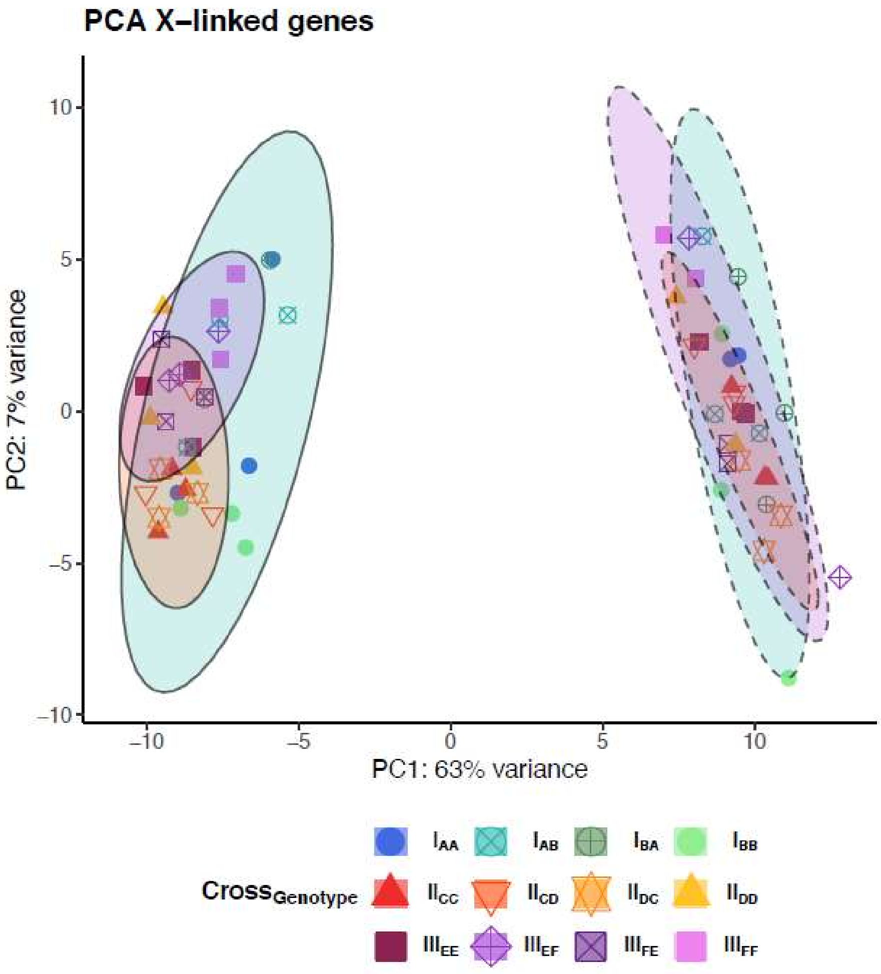

Fig. S4| Overview of variation in X-linked gene expression (PCA). The crosses (I, II, II) are indicated by colors and ellipses, the genotypes with symbols. Female and male samples are shown in solid and dashed ellipsoid outlines, respectively. As for the autosomal expression, samples cluster well by sex along PC1 (indicated by the separation of the ellipses), explaining a large part of variance in expression (63%). The PCA is based on those N = 474 X-linked transcript (out of a total of 1841 X-linked transcripts) that passed the pre-filtering steps. Ellipses represent data ellipses (95% of expected data under a bivariate normal distribution).

.

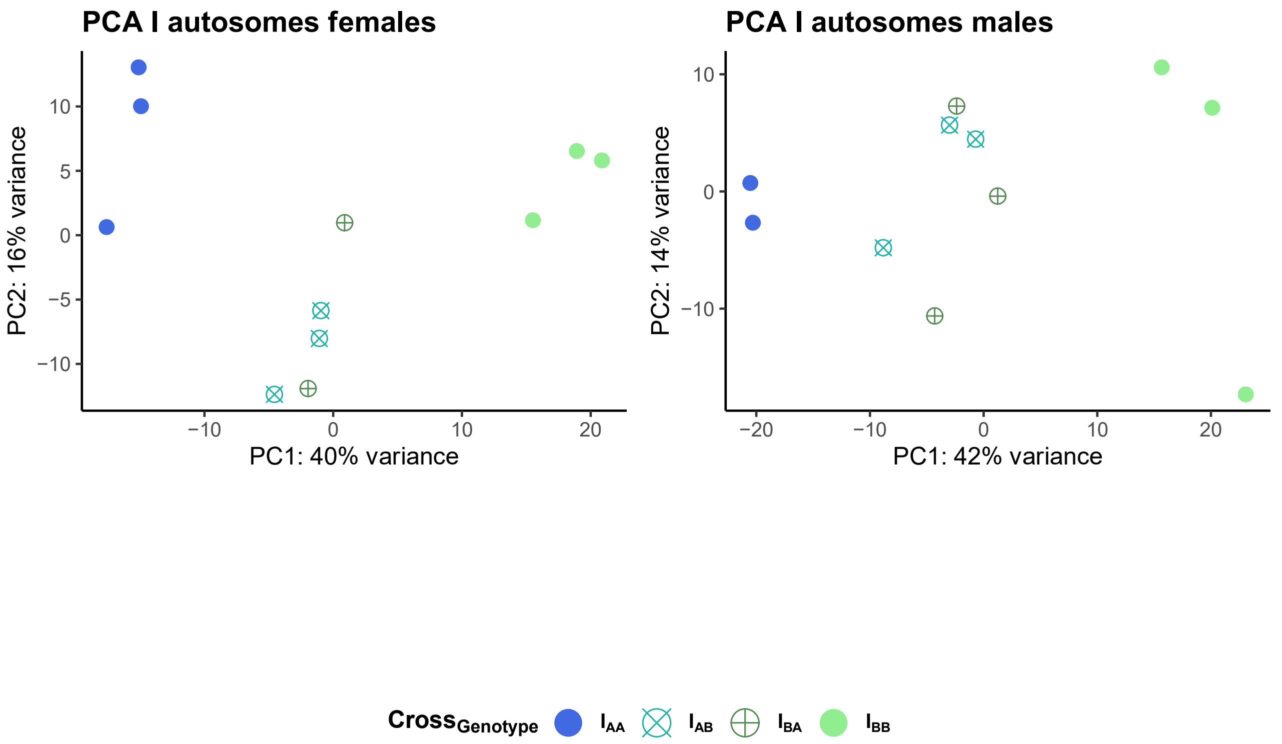

Fig. S5| Cross I: autosomal transcript expression (PCA). In both females and males, samples cluster by genotype on PC1, where heterozygous F_1 offspring (open circles) are in between the homozygous parental samples (filled in circles). PCA is based on N = 12010 autosomal transcripts.

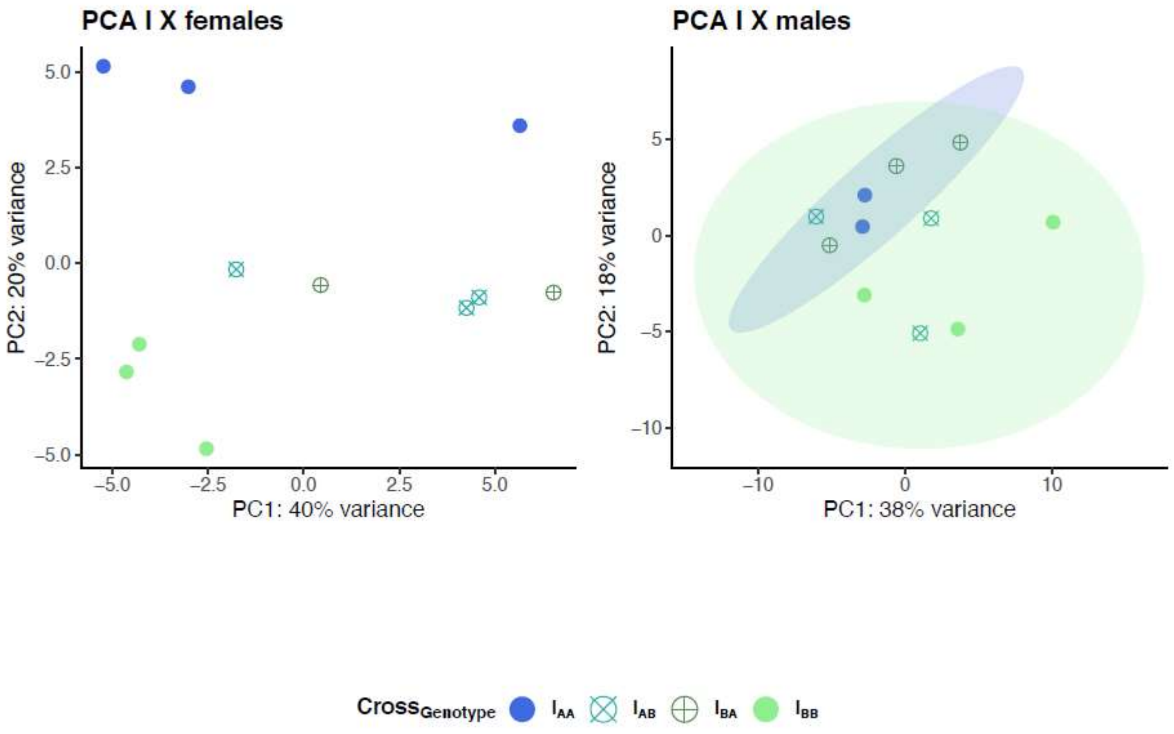

Fig. S6| Cross I: X-linked transcript expression (PCA). In females – similar as for autosomal transcripts – F_1 heterozygotes cluster in between the homozygous parental inbred lines on PC2. In males, where X is maternally inherited (i.e. X is haploid in males, hence hybrid males are hemizygous for X), F_1 males are expected to cluster by their mother's genotype (highlighted with ellipsoids). There is no clear pattern of clustering by the mother's genotype in male X-linked expression (N = 496 transcripts). Ellipses represent data ellipses (95% of expected data under a bivariate normal distribution).

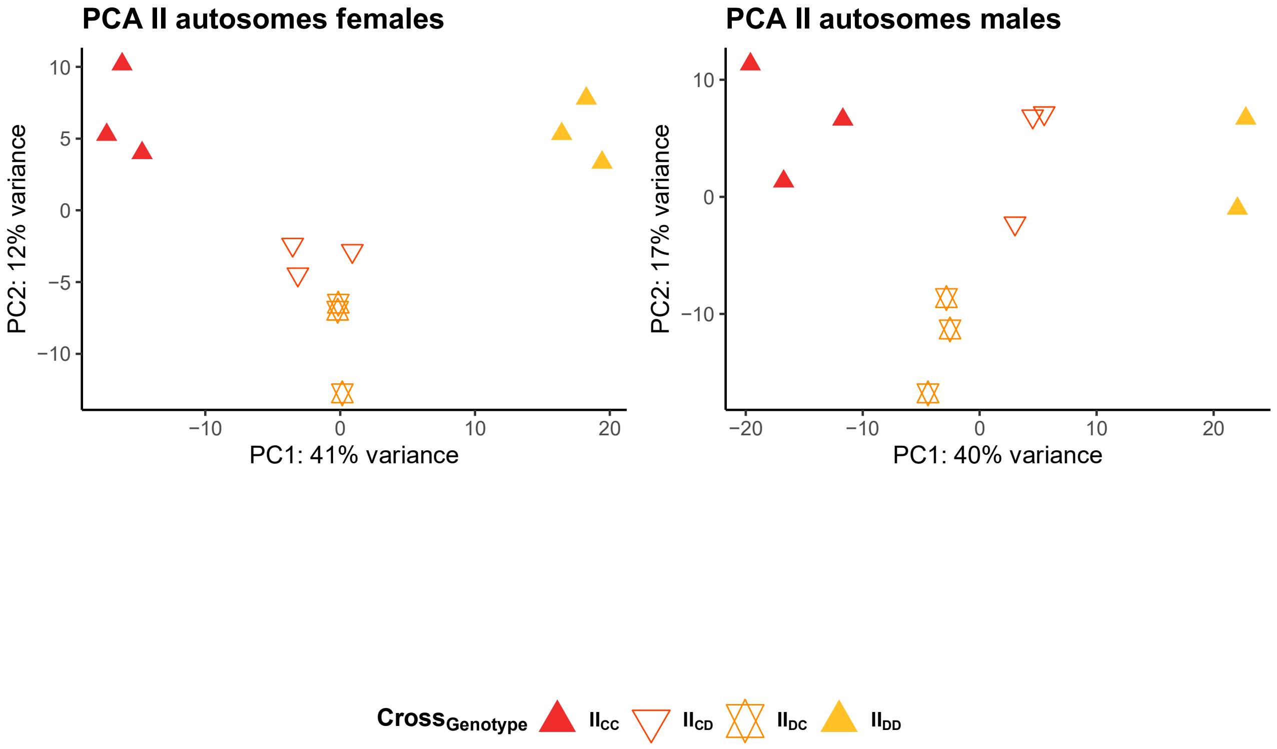

Fig. S7| Cross II: autosomal transcript expression (PCA). Samples cluster by genotype along PC1, where F_1 heterozygotes (open shapes) are intermediate to the two homozygous parental inbred lines (filled in shapes). Here, there is a tendency for a parent of origin effect in heterozygotes, which is accounted for in our analysis for dominance. N = 11455 transcripts.

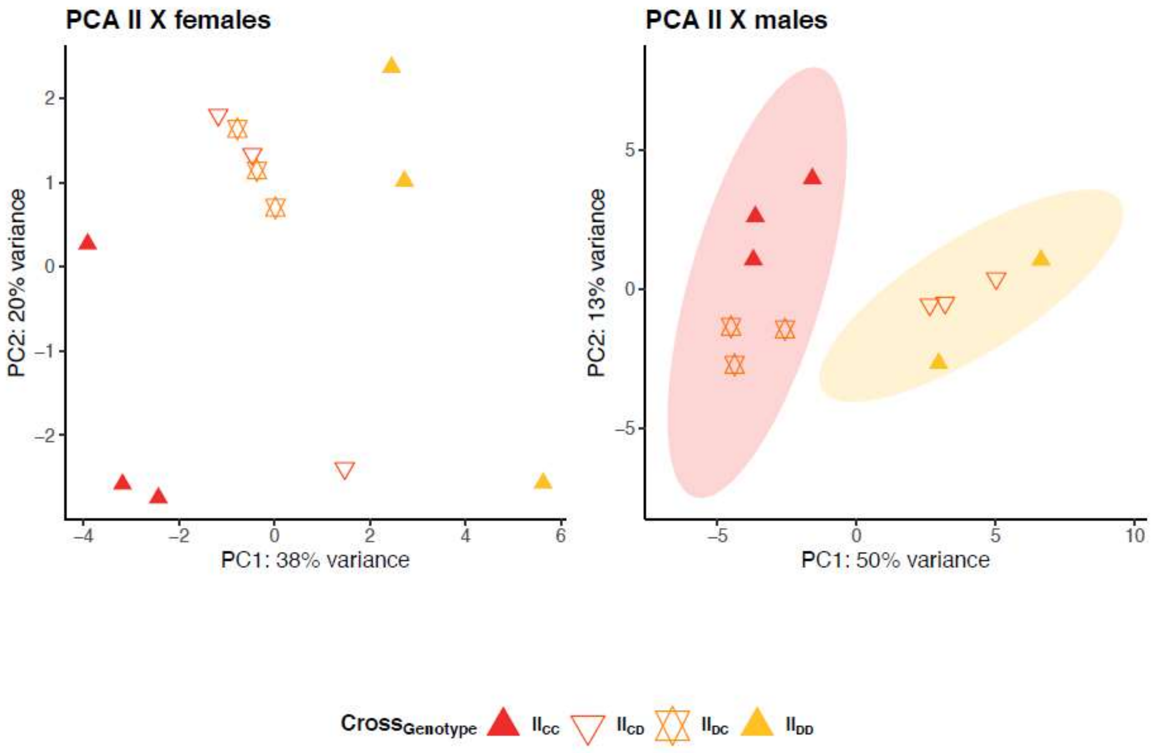

Fig. S8| Cross II: X-linked transcript expression (PCA). Female F_1 heterozygotes cluster in between the homozygous parental inbred lines. Here, F_1 males cluster by their mother's genotype (highlighted with ellipsoids). N = 496 transcripts. Ellipses represent data ellipses (95% of expected data under a bivariate normal distribution).

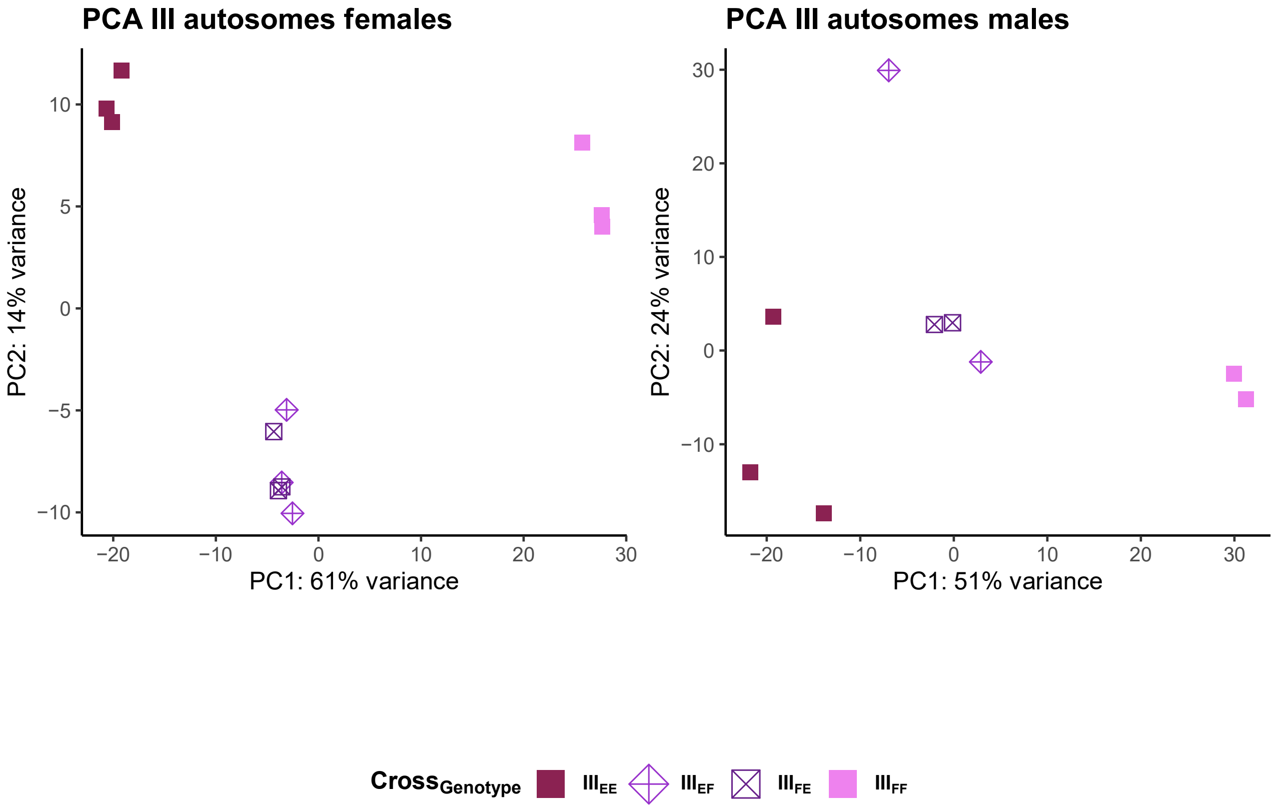

Fig. S9| Cross III: autosomal transcript expression (PCA). Both female and male samples cluster by genotype along PC1, and heterozygous F_1 offspring (open squares) are intermediate to the homozygous parental lines (filled in squares). N = 11812 transcripts.

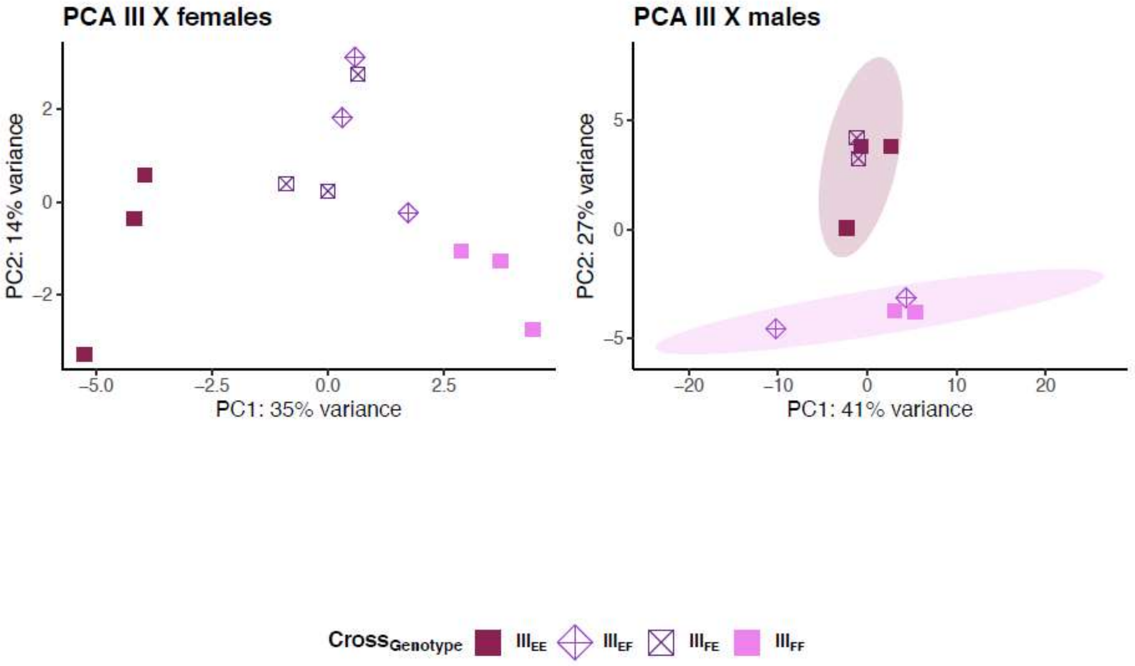

Fig. S10| Cross III: X-linked transcript expression (PCA). X-linked transcript expression in female F_1 heterozygotes cluster in between the homozygous parental inbred lines along PC1. Again, male F_1 cluster by their mother's genotype (PC2) (highlighted with ellipsoids). N = 507 transcripts. Ellipses represent data ellipses (95% of expected data under a bivariate normal distribution).

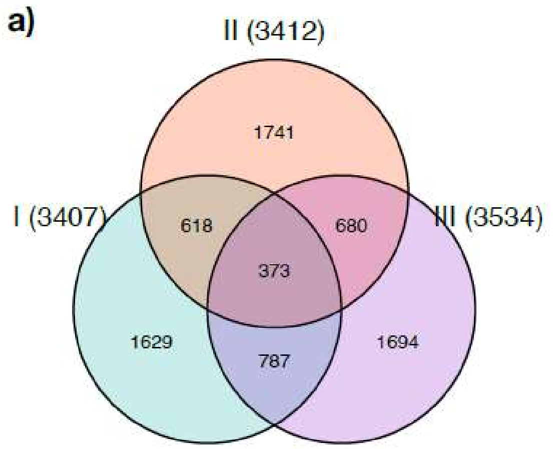

Fig. S11| Overlap of analyzed autosomal transcripts in the three crosses (I-III). **a)** Venn diagram of autosomal transcripts showing a sex concordant difference in expression between the sexes, in the two homozygous parental inbred lines (focal sets). **b)** Subset of autosomal transcripts that also show significant sex-specific dominance in the F_1 heterozygote crosses.

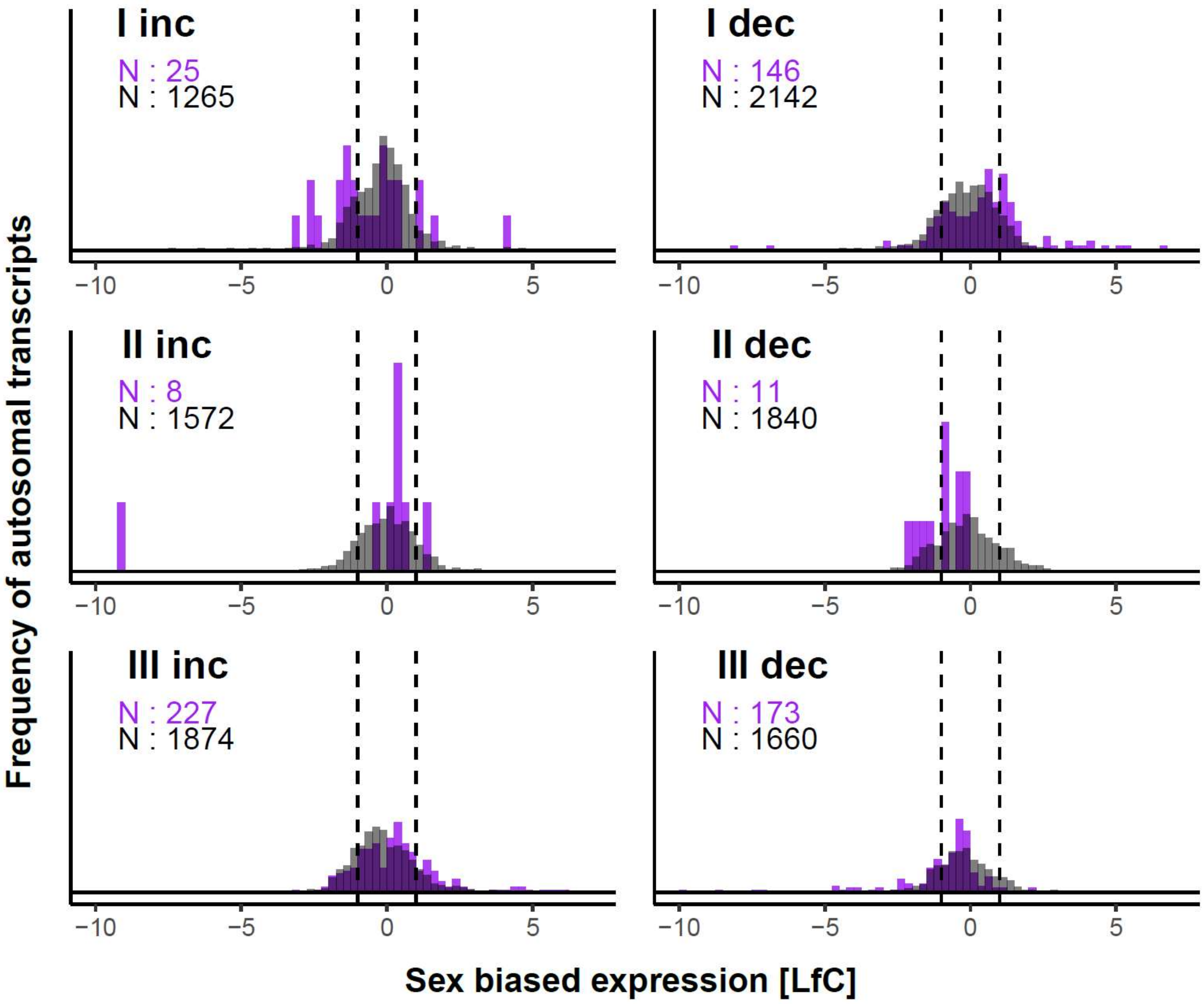

Fig. S12| The frequency distribution of autosomal transcripts (density histogram) that showed an increase (left panels) or a decrease (right panels) in the degree of sex biased expression in heterozygotes relative to the average in the two homozygotes, across the overall degree of sex biased expression. This figure illustrates the relative occurrence of the two possible cases illustrated in the right panels of Figure 1. The left three panels show cases corresponding to the scenario in the top right panel in Figure 1, and the right three panels to that in the lower right panel in Figure 1. The x-axis shows sex-biased expression as a log2 fold change (LfC). Negative values mean that a transcript is male biased in expression and positive that it is female biased. Dashed lines indicate a two-fold difference between the sexes in transcript abundance. The bars represent those transcripts from our focal gene set that either showed (purple) or did not show (grey) a significant (q = 0.05) sex × dominance interaction. Numbers represent the number of transcripts. Over all focal transcripts, a fairly similar proportion showed an increase (I: 36%, II: 46%, III: 53%) and a decrease (I: 64%, II: 54%, III: 47%) in sex biased expression in heterozygotes, relative to homozygotes. A three-way contingency table test (increase vs. decrease, significant vs. non-significant sex-by-dominance, cross type; a 2 × 2 × 3 log-linear model) showed that the proportion of transcripts showing increased over decreased sex bias in heterozygotes did not differ overall between those showing significant sex-specificity in dominance and those that did not (the interaction between increase/decrease and significant/non-significant: $G^2_1 = 0.46$, $p = 0.498$). This suggests that genes showing sex-specificity in dominance are similar in directionality compared to genes do not show sex-specificity in dominance. Yet, we note that this effect differed significantly across the three independent crosses (three-way interaction: $G^2_7 = 671.14$, $p < 0.001$) (see Results section for details).

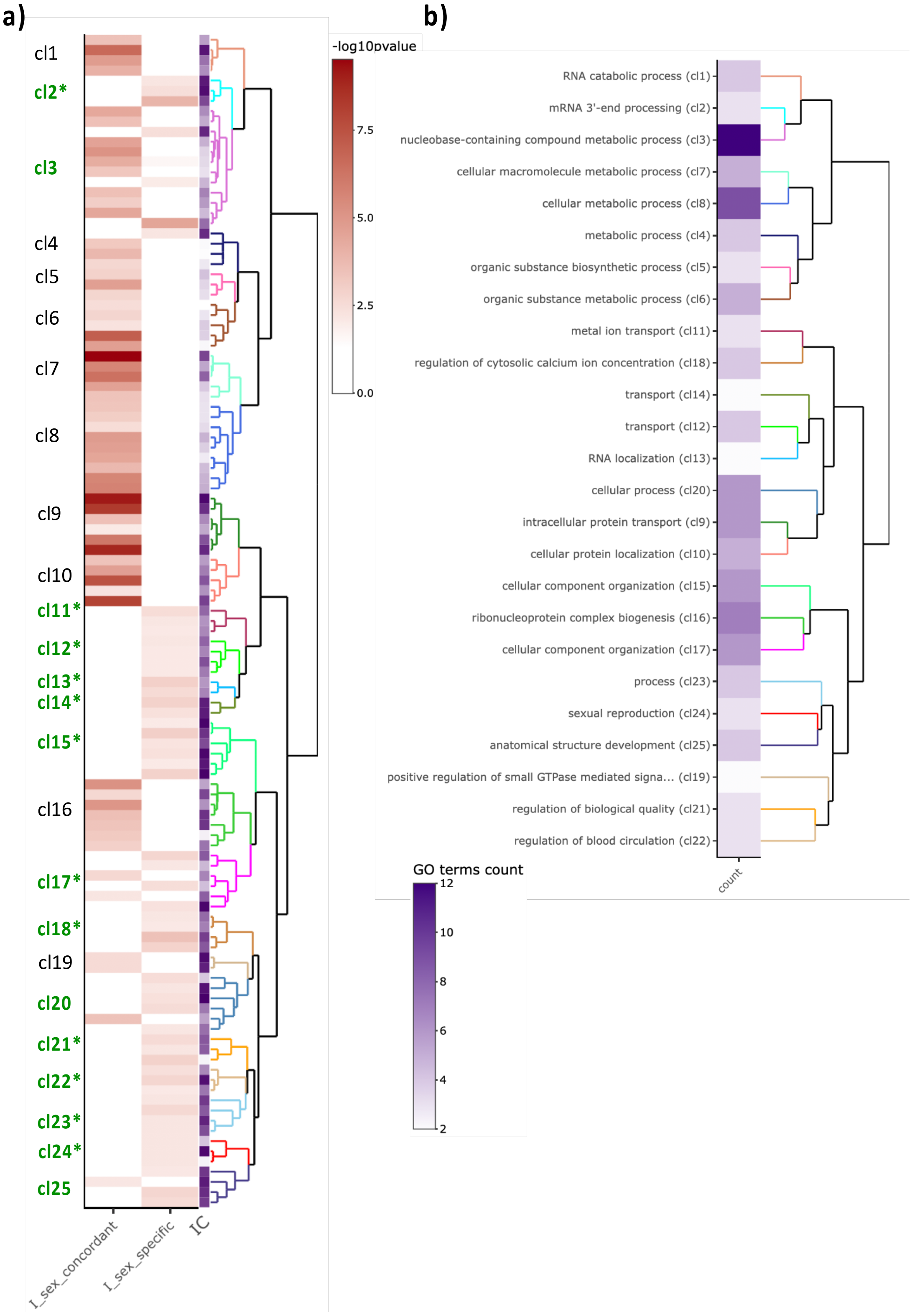

Fig. S13| Cross I gene ontology enrichment comparison. **a)** GO term dendrogram based on Wang's semantic similarity distance and ward.D2 clustering. Heatmap shows significance of the enrichment for genes with significant sex-concordant dominance (N = 263, left column) or sex-specific dominance (N = 171, right column) as compared to all genes that passed filtering in cross I (N = 3407). GO term clusters showing significant enrichment for genes with sex-specific dominance are highlighted in green, and additionally with an asterisk if they are only enriched for genes with sex-specific dominance but not for genes with sex-concordant dominance. **b)** Dendrogram based on best-match average semantic similarity and ward.D2 aggregation, showing the first common GO term ancestor for each cluster from (**a**) and the number of enriched GO terms within each cluster in purple.

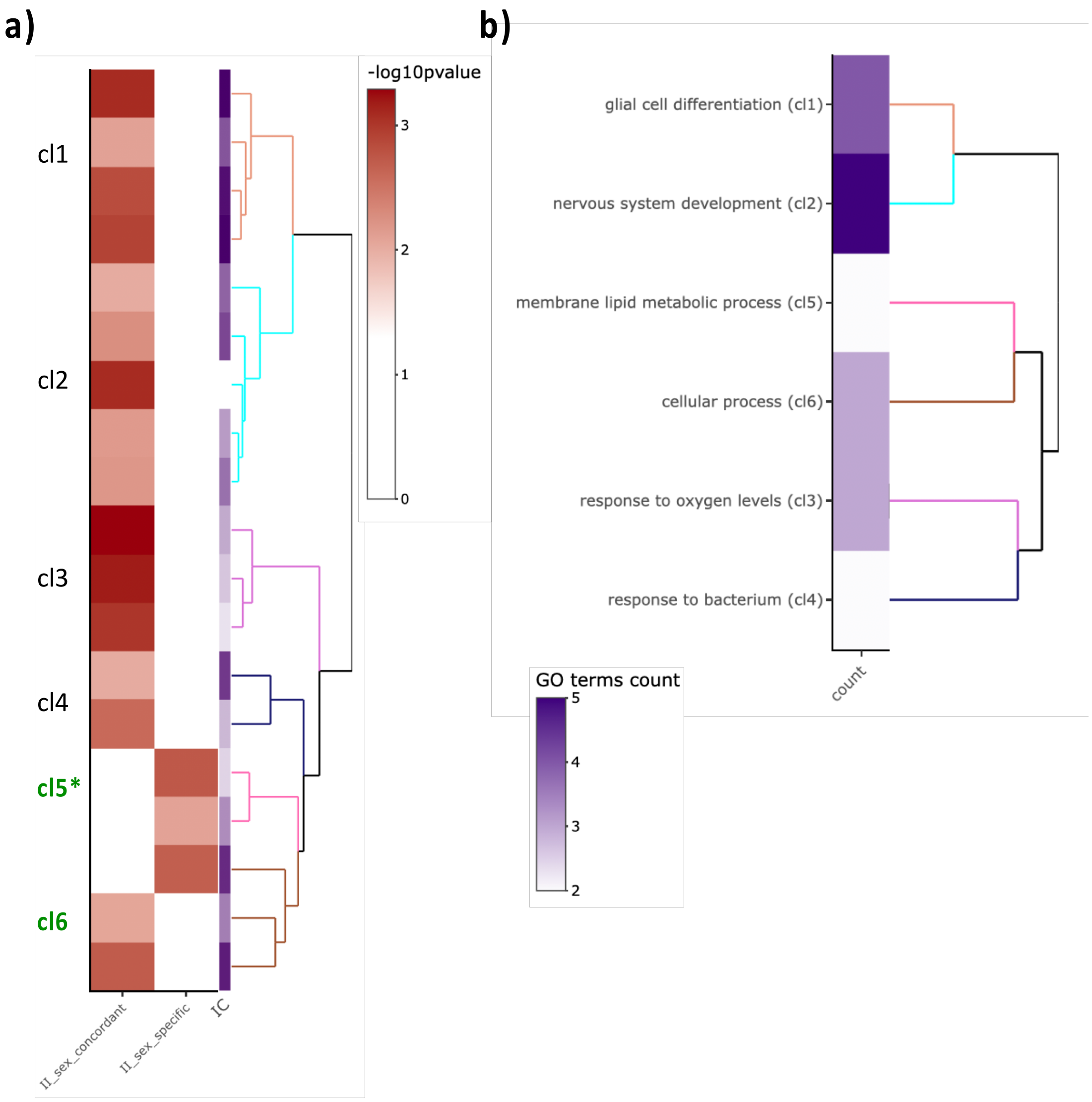

Fig. S14| Cross II gene ontology enrichment comparison. **a)** GO term dendrogram based on Wang's semantic similarity distance and ward.D2 clustering. Heatmap shows significance of the enrichment for genes with significant sex concordant dominance (N = 17, left column) or sex-specific dominance (N = 19, right column) as compared to all genes that passed filtering in cross II (N = 3412). GO term clusters showing significant enrichment for genes with sex-specific dominance are highlighted in green, and additionally with an asterisk if they are only enriched for genes with sex-specific dominance but not for genes with sex-concordant dominance. **b)** Dendrogram based on best-match average semantic similarity and ward.D2 aggregation, showing the first common GO term ancestor for each cluster from (**a**) and the number of enriched GO terms within each cluster in purple.

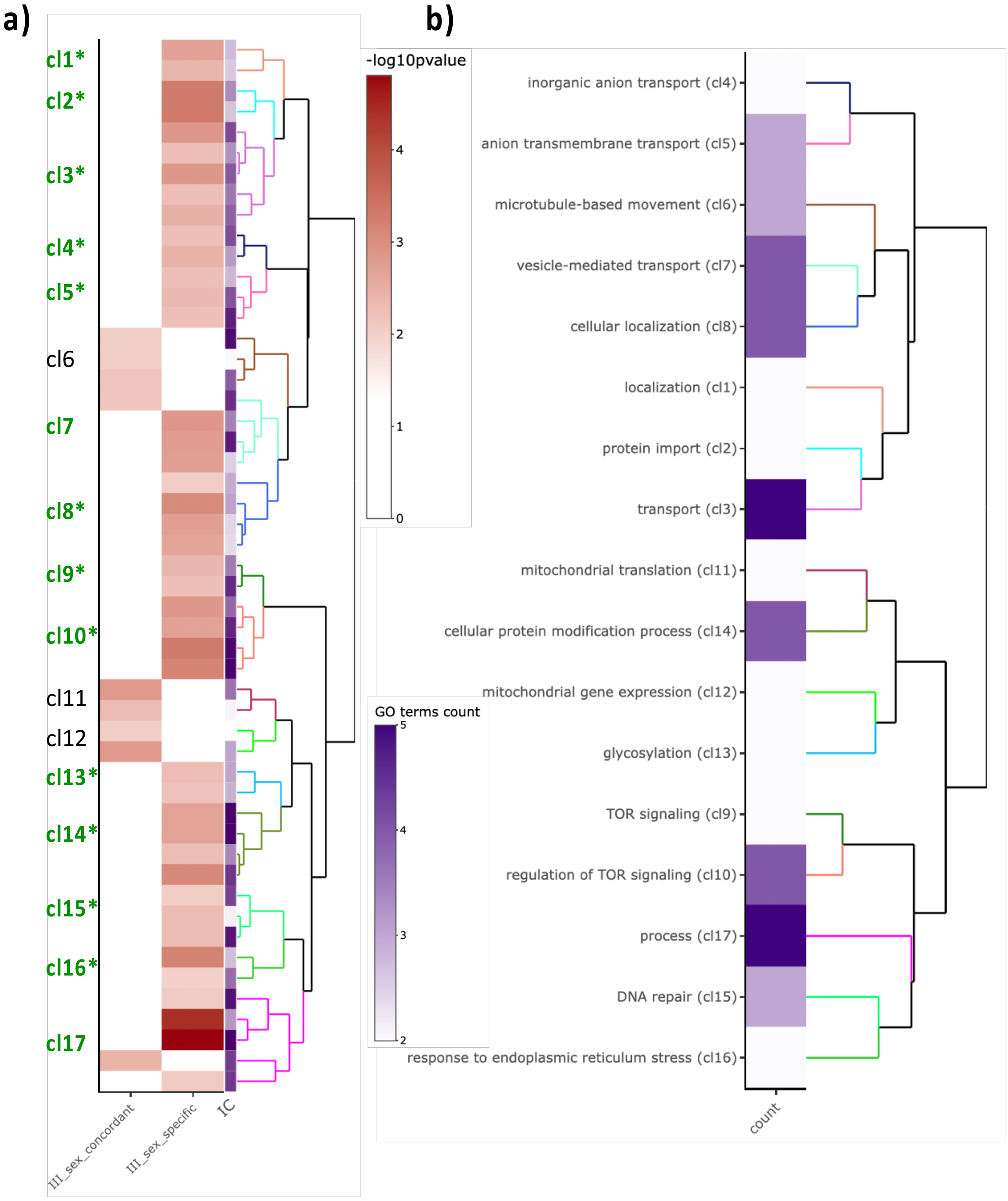

Fig. S15| Cross III gene ontology enrichment comparison. **a)** GO term dendrogram based on Wang's semantic similarity distance and ward.D2 clustering. Heatmap shows significance of the enrichment for genes with significant sex concordant dominance (N = 158, left column) or sex-specific dominance (N = 400, right column) as compared to all genes that passed filtering in cross III (N = 3534). GO term clusters showing significant enrichment for genes with sex-specific dominance are highlighted in green, and additionally with an asterisk if they are only enriched for genes with sex-specific dominance but not for genes with sex-concordant dominance. **b)** Dendrogram based on best-match average semantic similarity and ward.D2 aggregation, showing the first common GO term ancestor for each cluster from (**a**) and the number of enriched GO terms within each cluster in purple.

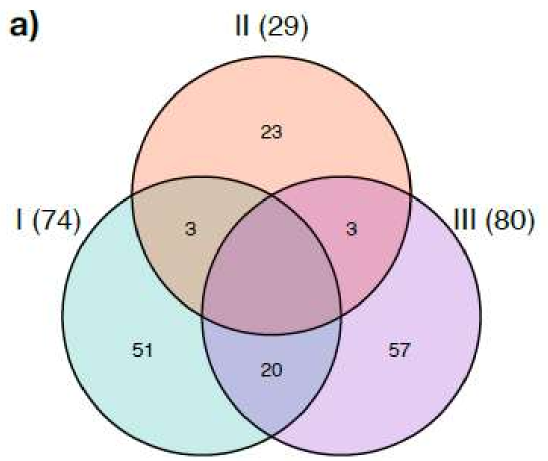

Fig. S16| Overview analyzed X-linked transcripts. **a)** Venn diagram of X-linked transcripts showing a sex-concordant difference in expression between the crossed homozygous parental inbred lines. **b)** Subset of X-linked transcripts that show significant sex-specific dominance in the F_1 heterozygotes.

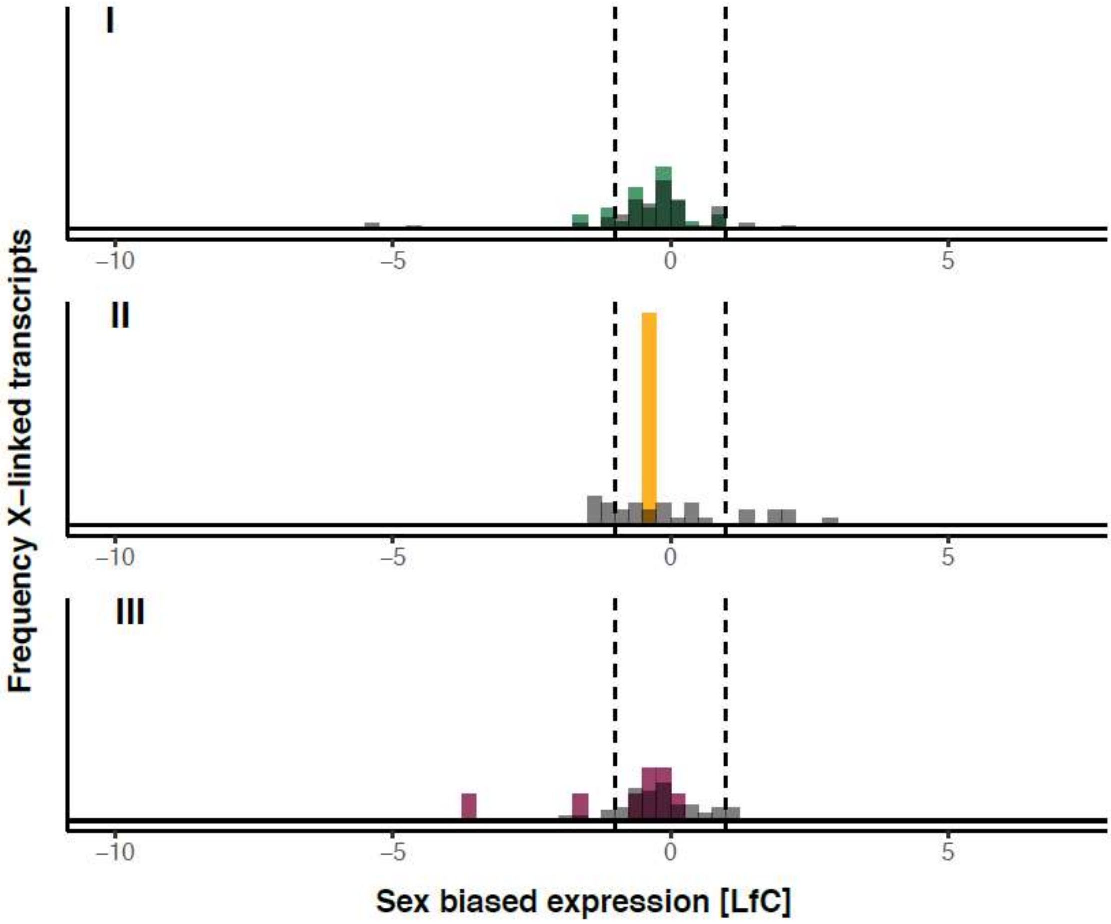

Fig. S17| Distributions of sex-bias in expression of X-linked genes. Density histograms of all considered transcripts serving as a reference distribution are shown in grey (focal sets) and density histograms of transcripts with significant sex-specific dominance (with q-value < 0.05) are color coded by cross. The x-axis shows sex-biased expression as a log2 fold change (LfC). Negative values mean that a transcript is male biased in expression and positive that it is female biased. Dashed lines indicate a two-fold difference between the sexes in transcript abundance.
